# Integrating Systems Biology and an *Ex Vivo* Human Tumor Model Elucidates PD-1 Blockade Response Dynamics

**DOI:** 10.1016/j.isci.2020.101229

**Published:** 2020-06-02

**Authors:** Munisha Smalley, Michelle Przedborski, Saravanan Thiyagarajan, Moriah Pellowe, Amit Verma, Nilesh Brijwani, Debika Datta, Misti Jain, Basavaraja U. Shanthappa, Vidushi Kapoor, Kodaganur S. Gopinath, D.C. Doval, K.S. Sabitha, Gaspar Taroncher-Oldenburg, Biswanath Majumder, Pradip Majumder, Mohammad Kohandel, Aaron Goldman

**Affiliations:** 1 Integrative Immuno-Oncology Center, Mitra Biotech, Woburn, MA, USA; 2 Department of Medicine, Harvard Medical School, Boston, MA, USA; 3 Division of Engineering in Medicine, Brigham and Women's Hospital, Boston, MA, USA; 4 University of Waterloo, Department of Applied Mathematics, Waterloo, ON N2L 3G1, Canada; 5 Max Super Speciality Hospital, New Delhi, India; 6 Bangalore Institute of Oncology, Bangalore, India; 7 Rajiv Gandhi Cancer Institute & Research Centre, New Delhi, India; 8 Kidwai Memorial Institute of Oncology, Bangalore, India; 9 Gaspar Taroncher-Oldenburg Consulting, Philadelphia, PA, USA

**Keywords:** Biological Sciences, Cancer Systems Biology, Immunology, Systems Biology

## Abstract

*Ex vivo* human tumor models have emerged as promising, yet complex tools to study cancer immunotherapy response dynamics. Here, we present a strategy that integrates empirical data from an *ex vivo* human system with computational models to interpret the response dynamics of a clinically prescribed PD-1 inhibitor, nivolumab, in head and neck squamous cell carcinoma (HNSCC) biopsies (N = 50). Using biological assays, we show that drug-induced variance stratifies samples by T helper type 1 (Th1)-related pathways. We then built a systems biology network and mathematical framework of local and global sensitivity analyses to simulate and estimate antitumor phenotypes, which implicate a dynamic role for the induction of Th1-related cytokines and T cell proliferation patterns. Together, we describe a multi-disciplinary strategy to analyze and interpret the response dynamics of PD-1 blockade using heterogeneous *ex vivo* data and *in silico* simulations, which could provide researchers a powerful toolset to interrogate immune checkpoint inhibitors.

## Highlights

• Computational strategy to study anticancer immune checkpoint blockade, *ex vivo*
• PD-1 blockade-induced T helper type 1 (Th1) stratifies tumor biopsies, *ex vivo*
• Systems biology links drug effect to dynamic intratumor T cell proliferation
• *In silico* sensitivity analyses of PD-1 blockade predict Th1-induced antitumor effects

## Graphical Abstract

**Figure:**
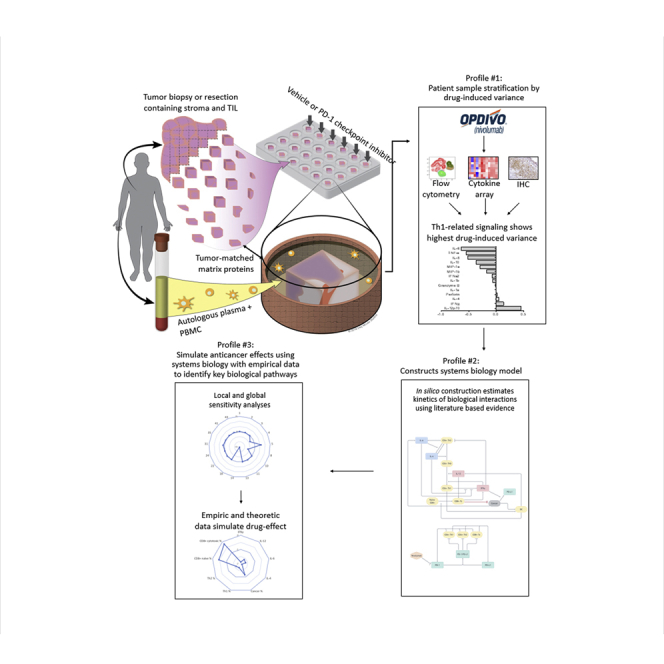

## Introduction

Cancer immunotherapies—therapies that harness the body's own immune system to fight cancer—have revolutionized cancer treatment over the past decade. A number of modalities, including immunomodulatory antibodies, adoptive immune cell transfer, and cancer vaccines have been clinically tested and brought to market. However, and despite their dramatic effect on survival rates and elimination of terminal disease in some patients, clinical success of cancer immunotherapies remains highly variable and notoriously unpredictable (Garon, 2017, Zhang and Chen, 2018). This variability and unpredictability of outcome is thought to be most likely driven by patient-specific biology (Kakimi et al., 2017, Wayteck et al., 2014), and in particular by interactions of the patient's immune system with the tumor (Spitzer et al., 2017). Predicting such interactions and studying them across heterogeneous tumors remains one of the biggest challenges in the space (Cristescu et al., 2018).

A key factor contributing to this state of affairs is a lack of well-established translational strategies and platforms that integrate inter- and intra- patient tumor heterogeneity, recapitulate cancer and stromal cell biology, recreate the tumor microenvironment and its underlying 3-dimensional architecture, and reproduce the immune compartment (Tannock and Hickman, 2016). Although current approaches to interrogate drugs, including *in vitro*, *in vivo*, and *ex vivo* preclinical models, have made great strides in addressing one or several of the above issues (Garnett et al., 2012, Samson et al., 2004, Sharma et al., 2010), most are limited by their inability to capture the full biological context of the native tumor at the individual patient level, which include the spatial arrangement of cell heterogeneity (Bertotti and Trusolino, 2013, Dhandapani and Goldman, 2017; Ruggeri et al., 2014, Samson et al., 2004). Indeed, *ex vivo* platforms are now routinely deployed to correlate empirical data with therapy response (Jahnke et al., 2014, Karekla et al., 2017, Silva et al., 2017). However, a paucity of literature has described meaningful analytical approaches to interpret intratumor immune biology with response dynamics of immune checkpoint blockade when clinical or therapy response is unknown. Indeed, such information could help fuel interrogation strategies and advance programs for pre-clinical investigation of cancer immunotherapy, such as checkpoint inhibitors.

**Figure 1:**
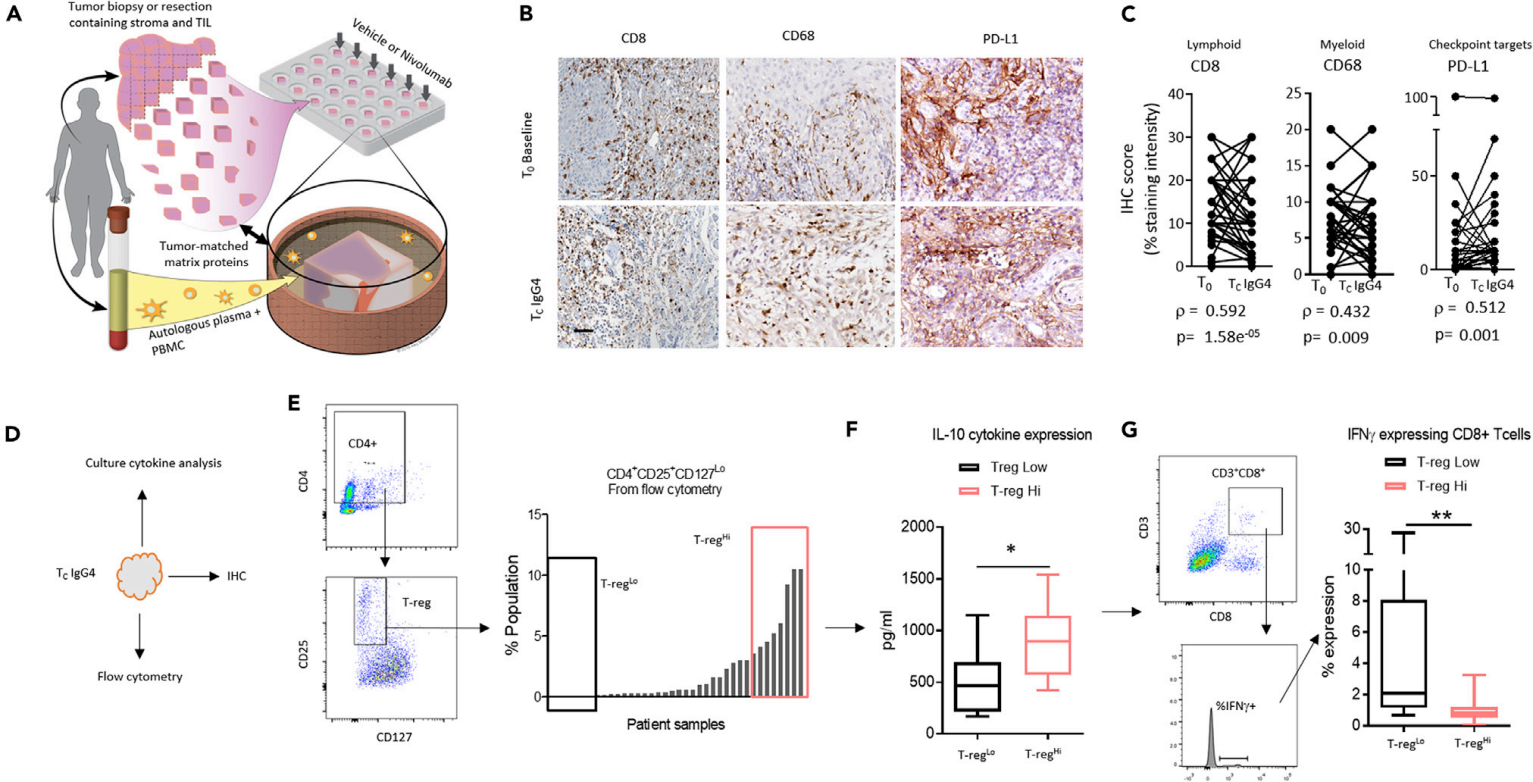
Profiling Spatiotemporal Immune Fidelity *Ex Vivo*, Comparing T_0_ with Unstimulated Vehicle Control (T_C_IgG4) (A) Schematic of the *ex vivo* tumor model. Surgically resected or biopsied tumor tissue is obtained along with patient-matched whole blood (i.e., time 0 h, T_0_). Following manual fragmentation, tissue is plated into individual tissue culture wells coated with indication- and grade-matched tumor matrix proteins along with autologous serum and peripheral blood mononuclear cells. Vehicle control or nivolumab was introduced to culture and interrogated for either 48 or 72 h (T_c_). Illustration by Wendy Chadbourne, 2018, Inky Mouse Studios, www.inkymousestudios.com. (B) Representative bright-field image from immunohistochemistry of three unique patient samples matching between T_0_ and T_c_. Scale bar, 40 μm. (C) Pairwise, Spearman correlation analysis was performed using IHC pathology scores of CD8, CD68, and PD-L1 between T_0_ and T_C_. Spearman rho was calculated to determine correlation between the two time points. p Value <0.05 indicates the correlation is statistically significant. (D) Schematic shows the different phenotypic response assays that are employed to study tumor phenotype and culture media during the *ex vivo* culture. (E) Flow cytometry was used to quantify the regulatory T cell (T-reg) population in all patient tumor samples. Right panel plots the percentage of T-regs in the total population. Boxes indicate the highest and lowest T-reg expressing patient samples (T-reg^Hi^ and T-reg^Lo^). (F) Box and whisker plot quantifies the IL-10 protein expression from the tissue culture media (pg/mL), determined by Luminex, in T-reg^Hi^ and T-reg^Lo^ patient samples (see [E]) ∗p < 0.05 by Mann-Whitney U test. (G) Box and whisker plot shows the percent expression of IFNγ in CD8^+^ T cells determined by flow cytometry in T-reg^Hi^ and T-reg^Lo^ patient samples, which were grouped from (E), ∗∗p < 0.01 by Mann-Whitney U test. See also Figure S1 contains patient demographic data.

We previously described a multi-compartment *ex vivo* platform, which preserves the cellular architecture and heterogeneity of solid tumors with a high degree of morphologic and kinase signaling fidelity (Majumder et al., 2015). The platform incorporates autologous peripheral constituents including immune cells and the patient's autologous plasma, which are explanted into a culture well containing tumor matrix proteins that match the grade or stage, and indication of each tumor type. To this, anticancer drugs are introduced to the co-culture for up to 3 days (Figure 1A). The utility of this platform for interrogating the biology of emerging cancer immunotherapies has yet to be tested, which requires interrogation of the immune compartment including a compatible and comprehensive analytical strategy to interpret the data.

Nivolumab (Opdivo) is one of two predominant US Food and Drug Administration-approved immune checkpoint inhibitors that targets programmed cell death protein 1 (PD-1). Pharmacodynamics (response dynamics) of PD-1 inhibitors are poorly understood, and therapy response to PD-1 inhibitors vary dramatically from patient to patient. The most widely explored biomarkers for predicting responders to PD-1 inhibitors are the expression level of programmed death-ligand 1 (PD-L1) and tumor mutational burden (TMB), which track to overall clinical response rates of 27% and 58%, respectively (Ferris et al., 2018, Goodman et al., 2017). Despite these advances, PD-1 inhibitors are still prescribed for patients with low or negative PD-L1 levels or low TMB because positive clinical benefit to anti-PD-1 drugs remain better when compared with chemotherapy (Ferris et al., 2018, Goodman et al., 2017). It is increasingly clear that a robust approach to study and interpret response dynamics of immune checkpoint inhibitors using completely human models may shift the course of drug development and our understanding for the mechanisms that confer response or resistance in the clinic.

Here, we describe a multi-pronged strategy to interrogate the response dynamics of the PD-1 checkpoint inhibitor, nivolumab, using our *ex vivo* system with human head and neck squamous cell carcinoma (HNSCC) biopsies (N = 50) where actual clinical response is unknown. First, we explored the fidelity of the platform for interrogating immuno-oncology drugs by establishing spatial distribution of immune cells and functional tumor-immune biology over the course of culture and examined these features across the lymphoid and myeloid compartments. Second, we describe a stratification method that considers patient-wide heterogeneity integrating drug-induced variance to compare the effect of nivolumab in subgroups of tumor samples with shared response dynamic profiles. Third and finally, we describe the use of a systems biology framework and mathematical simulations of local and global sensitivities to estimate the contribution of starting and drug-induced values from the empirical data as they impact “anti-tumor” effects. The approach outlined here provides both an unbiased picture for the downstream effects of PD-1 checkpoint blockade and an interdisciplinary analytical methodology to interrogate response dynamics from heterogeneous *ex vivo* data, which could be applied to other similar pre-clinical cancer immunotherapy models.

## Results

### Testing for Preservation of the Tumor-Immune Contexture, *Ex Vivo*, across Multiple Biological Assays

We deployed an *ex vivo* tumor culture system comprising live tissue fragments, which contain intact tumor, stroma, and infiltrated immune cells, as well as patient-autologous peripheral immune cells supplied in the culture media with plasma ligands (Figure 1A). We hypothesized that this system would provide a suitable substrate to interrogate rapidly induced intratumor response dynamics of immune checkpoint inhibitors. To test this hypothesis, we first explored fidelity of the tumor-immune contexture during *ex vivo* culture in unstimulated conditions (IgG4 vehicle control). We obtained tumor samples from patients with advanced and late stage HNSCC (Figure S1) and tested preservation of the tumor-immune microenvironment. Primarily, we examined retention of protein expression patterns, as well as lymphocyte infiltration and spatial heterogeneity between T_0_, which is defined as the time when the tumor arrives at the laboratory (24–36 h from resection or biopsy in the clinic), and T_C_, which is defined as the period 48–72 h after *ex vivo* culture. In this case, T_C_ is in the absence of exogenous stimuli (i.e., IgG4 control). First, we tested for retention of tumor-resident T-cells (CD8), macrophages (CD68), and tumor markers, such as PD-L1 over the course of the *ex vivo* culture. Using immunohistochemistry (IHC) and pathology scoring, we determined there was a high degree of concordance between T_0_ and T_C_ during culture, indicated by Spearman correlation (Figures 1B and 1C). In confirmation of these data, we analyzed tissue fragments by flow cytometry (Figure S2A) and quantified spatial arrangement of lymphocytes in the tumor versus stroma at both T_0_ and T_C_, detecting a similar degree of preservation (Figures S2B–S2D).

Next, we deployed multiple biological assays including flow cytometry of tumor tissue fragments, multiplex cytokine analysis of the tissue culture supernatant, and IHC to ask whether expected biological networks were retained post culture. First, as a cross-technology validation, we confirmed that expression of CD8 in IHC overlapped with the expression patterns of CD8 in flow cytometry from the same patient samples (quantified as deviation from the mean), suggesting consistency across different assays performed (Figures S3A and S3B). Next, we segregated patient samples based on expression levels of Foxp3 from IHC, a biomarker of immune suppressive T-reg cells, separating samples into two cohorts: high-expressing (Foxp3^Hi^) and low-expressing (Foxp3^Lo^). We confirmed cohort membership by showing the Foxp3^Hi^ subset contained significantly more Foxp3^+^ T-reg cells compared with the Foxp3^Lo^ cohort (p < 0.05) as determined by a different biological assay, flow cytometry (Figures S3C and S3D).

Finally, we tested whether we could recapitulate *in vivo* signaling mechanisms that contribute to lymphocyte lineage differentiation. For example, microenvironments enriched for T-reg cells are also enriched for IL-10 cytokines and often inversely correlate to the abundance of IFNγ+ CD8+ T cells (Saraiva and O'Garra, 2010). We used flow cytometry to first segregate the biopsies into two cohorts—high T-reg (T-reg^Hi^) and low T-reg (T-reg^Lo^) abundance—based on CD4^Hi^, CD25^Hi^, and CD127^Lo^ expression (Figures 1D and 1E). We confirmed the relationship between IL-10 expression and T-reg abundance, which was significantly higher in the culture supernatant of T-reg^Hi^ tumor samples than in the T-reg^Lo^ ones (p < 0.05) (Figure 1F). As expected, IFNγ+ CD8+ T cells were significantly lower in the T-reg^Hi^ tumor samples than in the T-reg^Lo^ ones (Figures 1F and 1G). Taken together, these data describe the level to which the *ex vivo* tumor culture preserves the tumor-immune contexture including biological networks and cross-assay fidelity.

### PD-1 Blockade-Induced Variance Identifies T Helper Type 1 as Conferring the Greatest Impact on Patient-to-Patient Heterogeneity

*Ex vivo* human tumor models pose a unique challenge because of the interpatient and intratumor heterogeneity. Thus, interpreting the data from *ex vivo* and organoid clinical explant models in the context of cancer immunotherapies remains a major challenge in cancer research (Dhandapani and Goldman, 2017, Maciejko et al., 2017).

**Figure 2:**
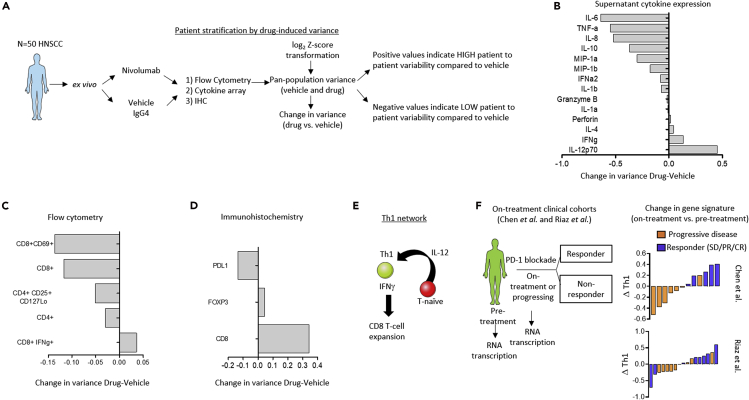
Drug-Induced Patient Variance as a Method to Stratify Heterogeneous Samples Pin Points a Role for the Th1-Related Pathway (A) Schematic shows analysis workflow to determine drug-induced variance. (B–D) Waterfall plots show the change in variance of cytokines, and gene and protein immune cell signatures in the vehicle control versus drug pressure from NanoString (A), flow cytometry (B), cytokine profiling (C), and immunohistochemistry (D). Calculation for variance can be found in the Transparent Methods section. Positive values indicate protein expressions that are more variable from patient to patient under nivolumab pressure compared with the vehicle control, i.e., the drug has the effect of creating high degree of phenotypic heterogeneity across all the patient samples. Negative values indicate those proteins signatures that are less variable across all patient samples under nivolumab pressure compared with the vehicle control, i.e., nivolumab has the effect of normalizing phenotype across patient samples relative to the vehicle. (E) Schematic shows the clinical study reported in Chen et al. and Riaz et al. (F) Waterfall plots show the measurable change of Th1 gene transcription signature in data obtained from Chen et al. and Riaz et al.

Given the range and diversity of phenotypes and drug-induced effects, we sought to deploy a method of stratifying the heterogeneous patient samples into smaller cohorts based on drug effect. To do this, we performed a variance calculation for control and treatment groups to detect the change in variance of protein expression patterns between samples after drug pressure. Data were transformed to log_2_ Z-scores to obtain mean of 0 and standard deviation of 1. The variance across all patient samples for each biomarker or signature within a treatment was calculated and vehicle variance was subtracted to obtain the change in variance. Using this method, we could determine whether the drug had a large impact on patient-to-patient response heterogeneity (i.e., positive change in variance) or whether the drug had little to no impact across patient samples compared with the vehicle control vis-á-vis a negative change in variance (Figure 2A). Using this strategy we determined that PD-1 blockade induced a high degree of interpatient heterogeneity in T helper type 1 (Th1)-related pathways indicated by the positive change in variance of IFNγ and IL-12 (Athie-Morales et al., 2004) cytokine expression levels and CD8^+^ IFNγ^+^ T cells (Ekkens et al., 2007) analyzed by flow cytometry, which was confirmed by IHC for CD8 (positive change in variance compared with the vehicle IgG4 control) (Figures 2B–2E).

To provide a translational impact to these findings, we obtained gene expression data from patients biopsied before treatment or while on-treatment, of PD-1 checkpoint therapy (Chen et al., 2016, Riaz et al., 2017) (Figure 2F) and examined Th1 gene expression profiles in the clinical dataset. We determined that a shift in the expression of Th1-related genes associated with better clinical response, as evidenced by the change in Th1 gene expression *Z* score in the waterfall plot (Figure 2F). Although this finding was expected, it supports the hypothesis that patient-to-patient drug-induced variability observed *ex vivo*, primarily in the expression of Th1-related phenotypes, may be a reasonable approach to stratify the heterogeneous *ex vivo* samples and study response dynamic profiles of the diverging subgroups, which may provide some information for features of “response” versus “resistance.”

**Figure 3:**
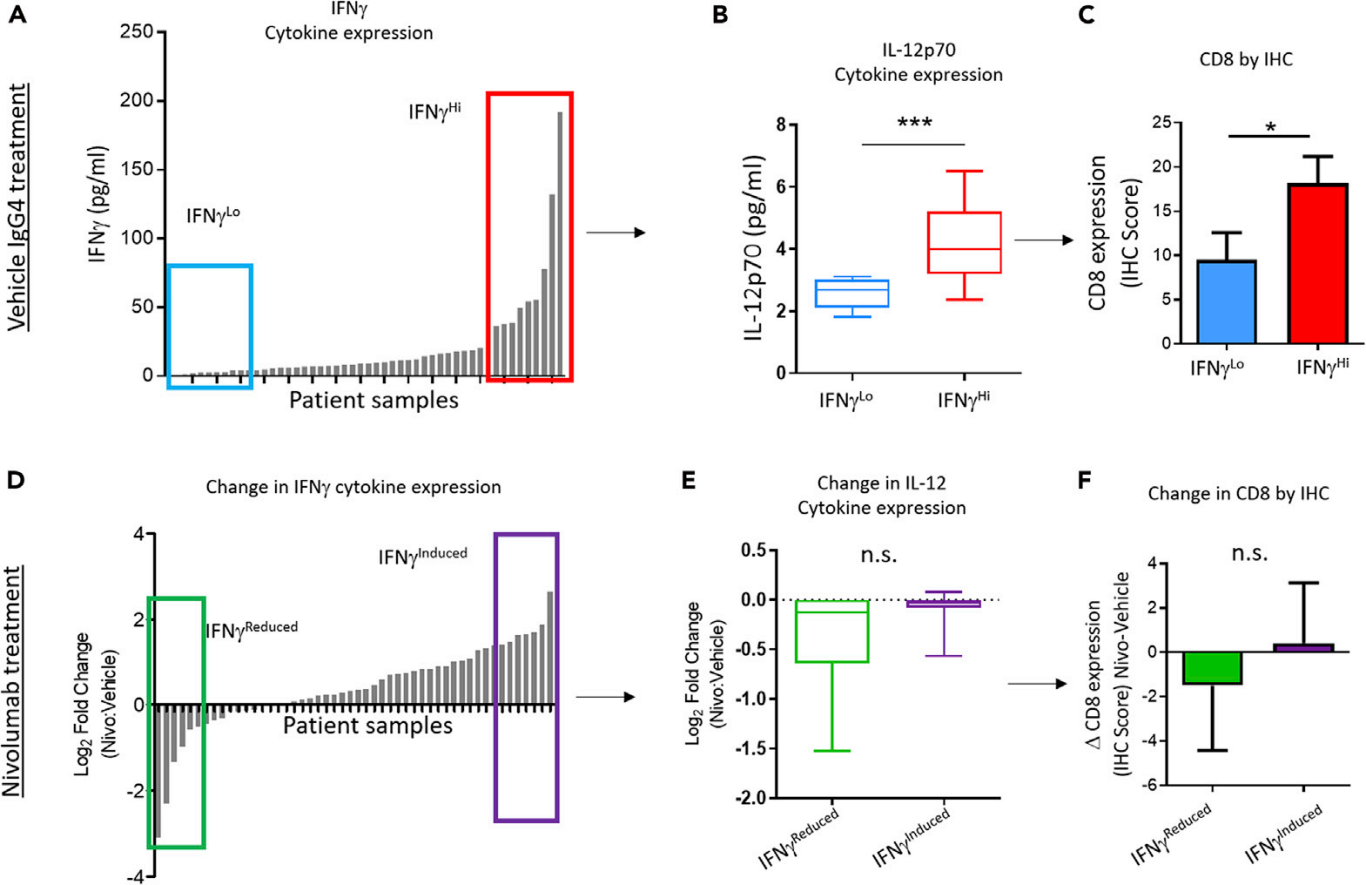
Th-1 Related Pathway Is Not Simultaneously Activated under Drug Pressure, *Ex Vivo* (A) Histogram shows IFNγ concentration (pg/mL) in the culture supernatant from the vehicle-treated cohort of all 50 patient samples determined as a mean expression at 24, 48, and 72 h culture. Boxes indicate patient samples that are stratified into the highest and lowest IFNγ expression (IFNγ^HI^ and IFNγ^Lo^). (B) Box plot shows IL-12p70 cytokine concentration in the culture media of IFNγ^HI^ and IFNγ^Lo^ cohorts, ∗∗∗p < 0.001 by Mann-Whitney U test. (C) Histogram shows expression of CD8 in tumor tissue of IFNγ^HI^ and IFNγ^Lo^ cohorts determined by IHC, ∗p < 0.05 by Mann-Whitney U test. (D) Waterfall plot shows log_2_ fold change in IFNγ concentration in culture media comparing nivolumab with vehicle IgG4. Colored boxes indicate the patient samples with the largest increase and decrease in IFNγ expression after PD-1 drug exposure (IFNγ^Induced^ and IFNγ^Reduced^, respectively). (E) Box plot shows IL-12p70 cytokine concentration in the culture media of IFNγ^Induced^ and IFNγ^Reduced^ cohorts, n.s. indicates sample sets are not significantly different by Mann-Whitney U test. (F) Histogram shows expression of CD8 in tumor tissue of IFNγ^Induced^ and IFNγ^Reduced^ cohorts determined by IHC, n.s. indicates sample sets are not significantly different by Mann-Whitney U test.

To this end, we asked whether expected biological pathways in the Th1 pathway were conserved or perturbed; we determined that, although expected biological networks, such as IFNγ, IL-12, and Th1 signaling cascade (Athie-Morales et al., 2004, Kieper et al., 2001) are retained in the vehicle control cohort (Figures 3A–3C), these same biological networks could not be recapitulated, or reasonably “linked together” after PD-1 blockade (Figures 3D–3F), which suggested that some biological mechanisms may not be able to be captured *ex vivo* in such a short culture period (i.e., up to 72 h) and therefore a more unbiased approach to stratify and study response dynamics should be employed for heterogeneous datasets.

### *In Silico* Simulation Implicates Dynamic Th1-Related Molecular Pathways in the Anticancer Effects of PD-1 Blockade

**Figure 4:**
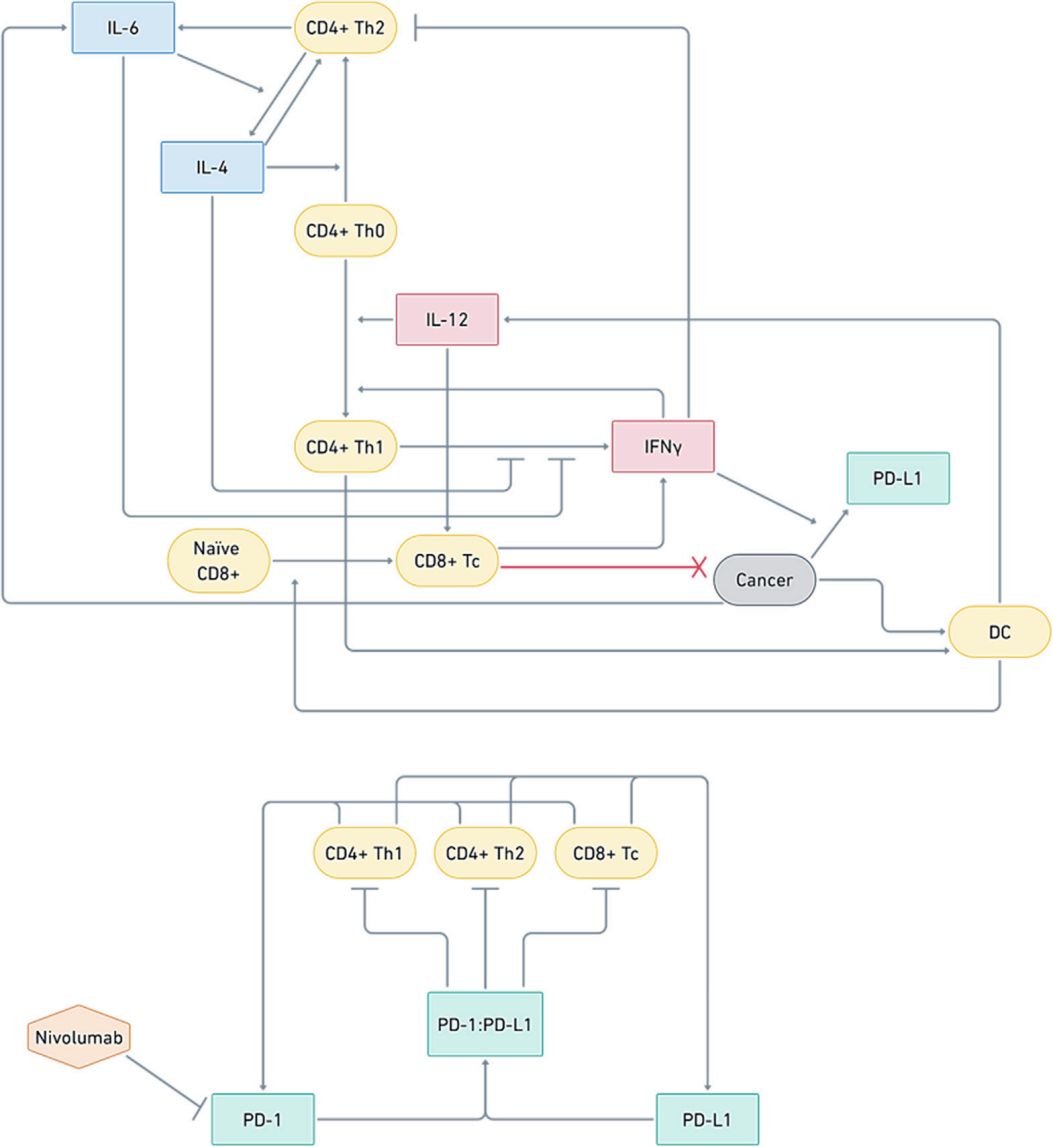
Systems and *In Silico* Strategy to Study Th1-Related Phenotypes in the PD-1/PD-L1 Network Systems biology model, illustrating interactions between cell populations, cytokines, and PD-1 and PD-L1. Naive CD4+ T helper cells (Th0) differentiate into CD4+ Th1 or CD4+ Th2 cells, which is influenced by Th1 cytokines (IL-12 and IFNγ) and Th2 cytokines (IL-4, IL-6). CD4+ Th1 cells influence the differentiation of naive CD8+ cells into CD8+ cytotoxic (Tc) T cells, which kill cancer cells. Cancer cells express PD-L1, which can bind to PD-1 expressed by CD4+ Th1, CD4+ Th2, and CD8+ Tc cells, thus inhibiting them.

**Figure 5:**
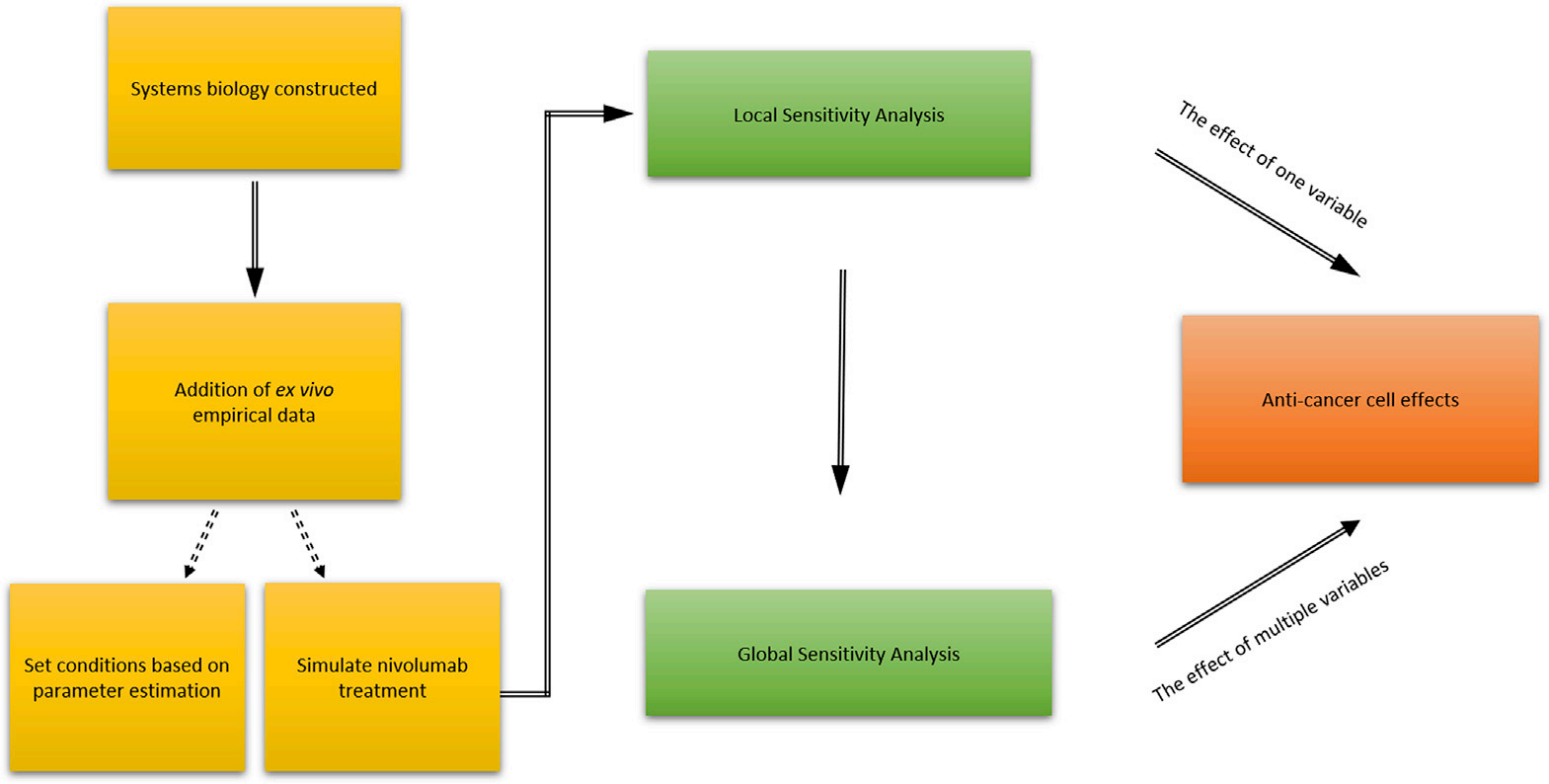
Integrating *Ex Vivo* Data into *In Silico* Analysis Schematic showing the procedure to integrate nivolumab-treated *ex vivo* empirical evidences *in silico* for local and global sensitivity analyses.

A major advance in the pre-clinical study of cancer immunotherapies is the integration of response dynamics with antitumor effects. Here, and in the absence of matched-patient clinical information, we wanted to infer how drug-induced response dynamics may link to putative anticancer effects of PD-1 blockade. We integrated the empirical *ex vivo* data, including initial starting concentrations and the dynamic range and changes of different Th1-related cell types and cytokines, into an *in silico* model. First, we developed a systems biology network comprising a tumor cell population along with five key interacting T cell populations and four key cytokines involved in T helper cell differentiation and activation (Figure 4). We then performed numerical simulations to investigate the sensitivity of the model's response to initial conditions and parameter values. The model consisted of 17 coupled ordinary differential equations (ODEs), which describe the time evolution of the cytokine concentrations, T cell populations, tumor cell population, and PD1 and PD-L1 levels (and the interaction of the latter with nivolumab). The 17 ODEs were parameterized by 47 distinct kinetic parameters (Supplemental Information). Summarized by the schematic in Figure 5, we then developed simulations of local and global sensitivity analysis to infer the effect of PD-1 blockade, the role of Th1-related cytokines and cell markers, and antitumor phenotypes. To do this, we used the *ex vivo* data in the context of nivolumab to determine the values of the model parameters, which was done by setting the initial T cell populations to the average of all patients (vehicle IgG4) and by setting the initial cytokine levels to values within the range of average ± SD of all patients (vehicle IgG4). Then, we integrated the nivolumab-treated cytokine data at the 24-h intervals (72 h total culture) and the T cell populations from flow cytometry to develop both a local and global sensitivity analysis (Supplemental Information).

**Figure 6:**
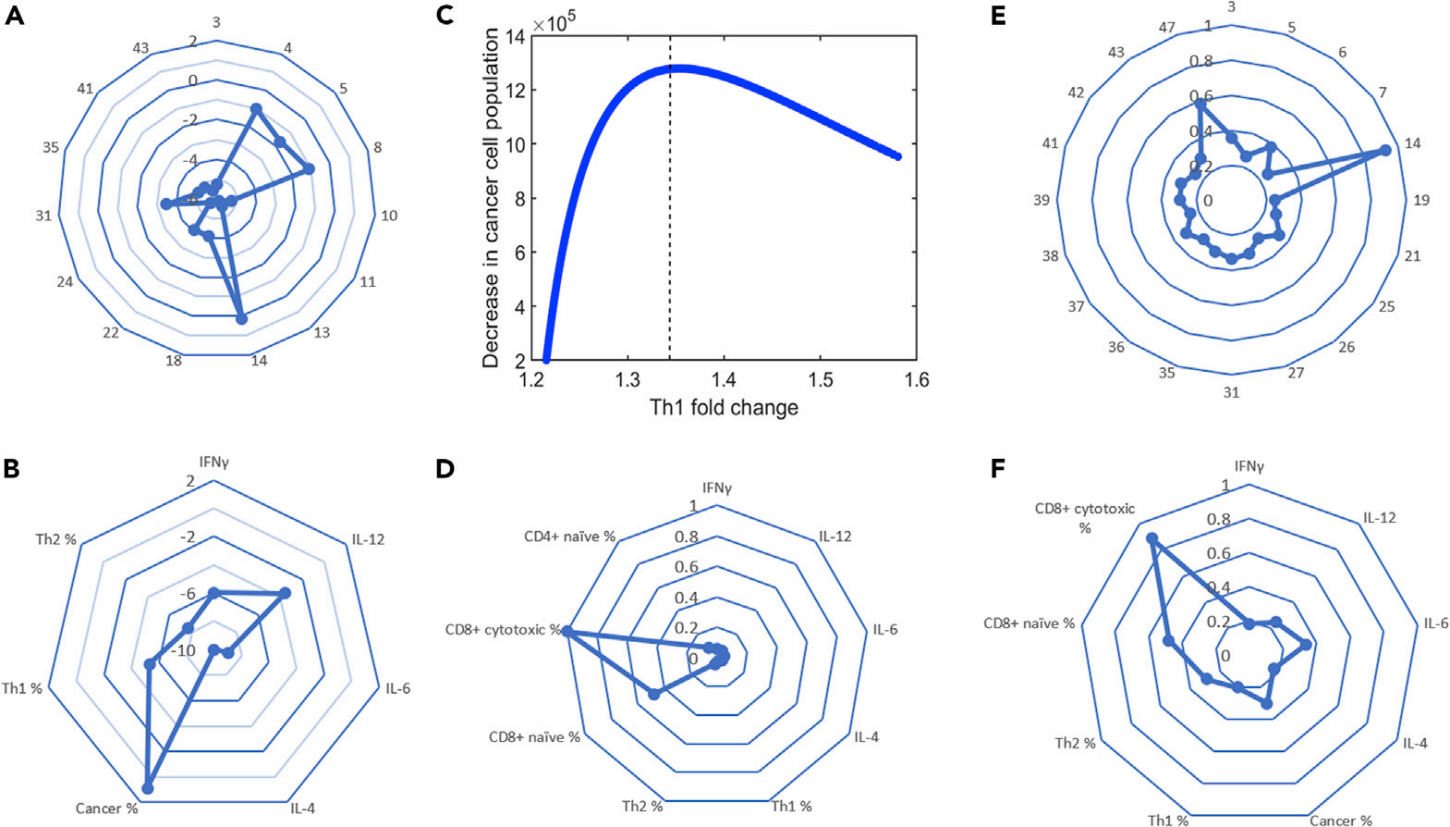
Local Sensitivity Analyses (LSA) and Global Sensitivity Analyses (GSA) Integrate Th1-Related Phenotypes to Simulate Antitumor Effect of PD-1 Blockade (A) Relative sensitivities determined by LSA for the top 15 kinetic parameters (indicated by parameter number). (B) Relative sensitivities determined by LSA for initial cytokine levels and initial T cell populations. For (A) and (B), the Log_10_ of the absolute value of the relative sensitivities are presented for visual clarity. (C) Decrease in cancer cell population at t = 72 h with PD-1 blockade as a function of Th1 induction, obtained by changing only the initial cancer cell population. Initial cancer cell population comprises less than 75% of the tumor for points to the left of the dashed vertical line. (D) MPSA sensitivities determined by GSA for the initial protein levels and initial relative T cell populations. (E) MPSA sensitivities determined by GSA for the top 20 kinetic parameters (indicated by parameter number). (F) MPSA sensitivities determined by GSA for the initial cytokine levels and T cell populations. In (E) and (F), all protein levels, initial T cell populations, initial cancer cell population, and kinetic parameters were varied.

Local sensitivity analysis was then conducted around the nominal parameter set to determine how small perturbations to the parameter values affect the strength of the response (defined as the increased assumed death of cancer cells) to treatment with nivolumab by varying one parameter at a time. The resulting relative sensitivities indicated that the efficiency of cytotoxic T cells at killing cancer cells (parameter 14), as well as kinetic parameters related to the proliferation rate of the cancer cells and the CD8+ cytotoxic T cells (parameters 4, 5, and 8), had the highest sensitivities and thus the largest effect on the strength of the response to treatment (Figures 6A and 6B). In the radial plots, a higher sensitivity value is indicated by a larger distance from the origin (center of the plot). Thus, as Figure 6A shows, most of the parameters corresponded to a small sensitivity value, except for those emphasized above.

Although small perturbations to single kinetic parameters affected the strength of the response to treatment, they were not enough to change the nature of the response to the treatment. Thus, we next performed global sensitivity analysis, which involved randomly changing all the initial cytokine levels and/or initial T cell populations and/or values of the kinetic parameters of the model simultaneously.

We determined that varying all the initial protein levels was not sufficient to induce a “non-response” phenotype; however, the strength of the response to treatment (as indicated by the final cancer cell population size) showed a power-law dependence on the initial IL-12 level of the form ${\tilde{C}}_{72}\propto {\left[\text{IL}-12\right]}^{-\beta },\;\beta >0$, where ${\tilde{C}}_{72}$ is the size of the cancer cell population at t = 72 h. In the model, the production of Th1 cells is dependent on IL-12 levels, which indicates that, as observed experimentally, an increase in Th1 levels may correlate with a stronger response to treatment. To investigate this point further, we kept the kinetic parameters, initial protein levels, and initial relative T cell populations fixed at their nominal values and varied only the initial cancer population level. We found that, when the initial cancer cell population comprises less than approximately 75% of the tumor biopsy, increased Th1 levels correlate with stronger treatment response. This trend is likely driven by the production of IL-12 by dendritic cells, which are assumed to be proportional to the cancer cell population. However, when the initial cancer population level exceeds more than approximately 75% of the tumor biopsy, the complex interplay between Th1 and Th2 cytokines produced by the cancer cells ultimately leads to decreasing response to treatment, despite increasing Th1 levels (Figure 6C, right side of the dashed vertical line).

Although changing the initial proportion of cancer cells comprising the biopsy affected the strength of the response to treatment, it did not induce a non-response phenotype for the nominal parameter set, even when the initial cancer population comprised up to 90% of the tumor cell population. To induce a non-response phenotype, it was necessary to change the initial relative T cell populations. In particular, by varying both the initial protein levels and initial relative T cell populations, while keeping all other parameters fixed at their nominal values, we could induce a simulated non-response phenotype. In this way, we found that the size of the cancer cell population at t = 72 h showed the highest sensitivity to the initial CD8+ cytotoxic T cell level, followed by the initial naive CD8+ T cell level (Figure 6D).

Finally, in an attempt to capture the heterogeneity in patient tumor microenvironment and response to treatment, we varied all the kinetic parameters, initial cytokine and PD-L1 levels, and initial T cell levels simultaneously. We used multi-parametric sensitivity analysis (MPSA) (Cho et al., 2003; Hornberger and Spear, 1981, Zi et al., 2005), which evaluates the parameter (and initial condition) sensitivities based on Kolmogorov-Smirnov statistics, returning sensitivity values between 0 and 1. A larger parameter sensitivity indicates that the corresponding parameter variation has a large impact on the model output (Zi, 2011). The results are presented in Figures 6E and 6F. Based on the MPSA sensitivities, the treatment response is most sensitive to the following kinetic parameters and initial conditions: the efficiency of cytotoxic T cells at killing cancer cells (parameter 14), the initial cytotoxic CD8+ T cell level, the rate of production of IFNγ by cytotoxic T cells (parameter 47), the initial naive CD8+ T cell level, the IL-4-independent growth rate of Th2 cells (parameter 6), the net proliferation rate of Th1 cells (parameter 3), the initial IL-6 level, and the half-maximal IFNγ concentration for IFNγ-dependent differentiation of naive CD4+ T cells into Th1 cells (parameter 25). These results reinforce the experimental observation that the variability in patient response is connected to the upregulation of Th1 levels.

Taken together, we report the integration of biological and mathematical strategies to interpret the response dynamics of PD-1 blockade in heterogeneous solid human tumor biopsies where matched-patient clinical information is missing. This approach took into consideration biological fidelity of the *ex vivo* system, methods of stratifying samples to identify drug-induced variability and systems biology approaches that can subsequently simulate key pathways contributing to antitumor phenotypes.

## Discussion

Predicting clinical response to therapy is a “holy grail” in the quest for durable, sustainable cures for cancer. Numerous preclinical and translational methods, including *in vitro* and *ex vivo* models, have been developed in the past decade to help guide our understanding for the clinical activity of immunotherapy (Jenkins et al., 2018, Meijer et al., 2017). Indeed, syngeneic animal models, which contain a full immune complex, are used to study cancer immunotherapies in a pre-clinical context, yet they often fall short in recreating the human response to drugs and immunotherapies as they lack critical lymphocytes (Day et al., 2015). Organotypic tumor spheroid models, on the other hand, recreate murine drug responses (Jenkins et al., 2018). Indeed, three of the most important aspects of assessing drug response in immunotherapy have been recently suggested as (1) the native spatial arrangement of the immune cells (Yuan, 2016), (2) autologous factors to recreate the host environment (Dhandapani and Goldman, 2017, Jackson and Thomas, 2017), and (3) clinically relevant integration of data to correlate response dynamics with predicted success or failure of a drug (Meijer et al., 2017). However, there remains a limited understanding for the biological and translational interpretations of data arising from *ex vivo* cancer immunotherapy models. To this end, our work provides a methodology to study adaptive immune responses using multiple biological and computational approaches, which elucidate pathways and signatures at the protein level. *Ex vivo* tumor systems in collaboration with systems biology and computational models could therefore be a powerful toolset to investigate and understand “dynamic drug response” of human tumors.

Here, we described an analytical approach that leverages an *ex vivo* model to study phenotypic “reflex” to anticancer immune checkpoint inhibitors. Importantly, we described preservation of the spatial tumor-immune contexture and conservation for the complex signaling networks between immune and tumor cells using autologous factors, which are unique to each patient. One question that remains open is the role that systemic lymphocytes (i.e., PBMCs) contribute to the *ex vivo* culture system. In a separate set of experiments using breast cancer samples, we determined that PBMCs will infiltrate the tumor fragment at a rate of 1%–2% of the total tumor CD45+ population, which becomes more variable when PD-1 checkpoint inhibitors are added (data not shown). This observation leads us to the conclusion that PBMCs may influence the spatial arrangement of immune cells in the tumor fragment and alter the immunobiology in response to PD-1 blockade. Indeed, in the present study induction of T helper cells, particularly Th1, was a putative indication for the conversion of an immune-deficient tumor into one that exhibited multiple inflammation-like features, including induction of pro-inflammatory cytokines. A more complete interrogation, in a separate study, is worthwhile in order to understand how exogenous immune cells influence the drug response, *ex vivo*.

Identifying checkpoint inhibitor-induced cell death in human *ex vivo* models is undescribed and remains a challenge in this space. In a study published by Jenkins et al., they used mouse-derived organotypic tumor spheroids (MDOTS), showing that immune-mediated cell death can be observed in a time frame of 5–6 days (Jenkins et al., 2018). However, in the same study, the observations were not recapitulated in a similar time frame using patient-derived organotypic tumor spheroids (PDOTS). Interestingly, Jenkins et al. described a change in the immune biology after exposure to PD-1 inhibitors, vis-á-vis changes to the cytokine expression profile within 72 h. This is not different from our findings. Indeed, we demonstrate that, in the absence of obvious cell death signals after treatment with PD-1 blockade in human samples, we do observe changes to the immune biology. Importantly, we demonstrate, for the first time, how this information can be leveraged with computational models to estimate the antitumor effects *in vivo*. Such an approach could help researchers understand anticancer effects of immunotherapy, leveraging changes to the immunobiology in the absence of obvious cell death markers.

Translational tools that recapitulate the human microenvironment are urgently needed to advance cancer research and drug development, particularly in the era of immunotherapy (Dhandapani and Goldman, 2017, Jackson and Thomas, 2017). While interrogating the effect of PD-1 blockade, we used an unbiased approach to dissect the response dynamics to PD-1 inhibition. Our expectation that we would be able to link known biological pathways to one another under drug pressure (e.g., IFNγ, IL-12, CD8+ T cell expansion) was thwarted when we applied nivolumab and attempted to elucidate molecular biology via IFNγ induction. Instead, we concluded that, although established biological mechanisms are preserved in the vehicle control groups, interpatient heterogeneity—and likely also the result of time in culture—confounded our ability to recapitulate known lymphocyte lineage differentiations and signaling relationships. For this reason, we determined that a more useful strategy was to examine drug-induced changes in variance across patient samples and use that information to guide a response dynamics approach, first. Subsequently, features of response and resistance could be bridged with response dynamics and drug-induced interpatient heterogeneity using variance of immune markers. In taking such an unbiased, integrative approach we successfully recapitulated biological features that have been previously described in the literature (e.g., acute phase reaction cytokines and induction of Th1-associated genes).

To estimate the anticancer effects of PD-1 blockade based on changes to immunobiology, we integrated the empirical *ex vivo* data into an *in silico* model. We first determined a set of nominal parameter values to match the average patient data, then we performed local and global sensitivity analysis to elucidate the parameters that were most important for influencing the treatment response. We found that local sensitivity analysis, in which the parameters are perturbed *individually* by small values around one parameter set, did not capture the variability in the untreated patient data. Since biological model inputs such as kinetic parameters and initial concentrations are thought to vary within a large range in different cell types and cellular environments (Zi, 2011), and are therefore expected to be highly variable between patients, it was necessary to vary several parameters simultaneously to capture the experimentally observed variability in treatment response. Importantly, in doing so, the *in silico* model reproduced the experimental observation that Th1 induction correlated with increased treatment response (under certain initial conditions). It should be noted that, although these results agree with experimental observations, only a subset of immune cell populations were included in the model and several simplifying assumptions were made to reduce the complexity of the model. In future work, we will relax some of these assumptions and include additional cytokines and immune cell populations and investigate potential resistance mechanisms to PD-1 blockade. Overall, these *in silico* data demonstrate the complexity of response dynamic changes that occur under nivolumab pressure, thus emphasizing the need for integrating multiple parameters profiled in an *ex vivo* model to inform the effect of immunotherapy intervention.

### Limitations of the Study

Further prospective evaluation is necessary. For instance, human papillomavirus (HPV) positivity in patients with HNSCC has been shown to correlate with a survival advantage (Benson et al., 2014). The HPV status of the HNSCC samples in this study is unknown, but HPV infections have been described at relatively low frequency in the same demographic population that our samples were obtained (Southern India) (Bandhary et al., 2018). If known, HPV status could allow for a better segregation and understanding of PD-1 blockade response dynamics. In addition, our evidence that spatial heterogeneity can provide unique information about the drug response role of CD4+ and CD8+ T cells could be expanded to understand their localization within the tumor. For example, intratumoral and stromal lymphocyte heterogeneity could provide functional information in the context of other solid tumors such as breast (Mani et al., 2016). Future studies that integrate these types of analytical features, which can be associated with the same patient's response in the clinic, could provide novel information about the behavior of patient-specific response to PD-1 blockade.

### Resource Availability

#### Lead Contact

Further information and requests for resources, additional data and reagents should be directed to and will be fulfilled by the Lead Contact: Dr. Aaron Goldman (agoldman@bwh.harvard.edu).

#### Materials Availability

New materials were not generated in the course of this study.

#### Data and Code Availability

Code related to simulated treatment protocols for the *ex vivo* experiments with specified inputs and outputs, as well as comments throughout the code, can be found at https://github.com/mprzedborski/ex-vivo-PD1-blockade. Raw data used for analysis and simulations will be available upon request.

## Methods

All methods can be found in the accompanying Transparent Methods supplemental file.

## Supplemental Information

Document S1. Transparent Methods, Figures S1–S3, and Tables S1–S3

## Footnotes

Supplemental Information can be found online at https://doi.org/10.1016/j.isci.2020.101229.
