# Supplementary material for: Integrating Systems Biology and an Ex Vivo Human Tumor Model Elucidates PD-1 Blockade Response Dynamics
Source: iScience. 2020 Jun 2;23(6):101229. doi: 10.1016/j.isci.2020.101229 (PMC7303667; doi:10.1016/j.isci.2020.101229)
Supplement: Document S1. Transparent Methods, Figures S1–S3, and Tables S1–S3 [file mmc1.pdf]

iScience, Volume *23*

Supplemental Information

Integrating Systems Biology and an *Ex Vivo* Human Tumor Model Elucidates PD-1 Blockade Response Dynamics

Munisha Smalley, Michelle Przedborski, Saravanan Thiyagarajan, Moriah Pellowe, Amit Verma, Nilesh Brijwani, Debika Datta, Misti Jain, Basavaraja U. Shanthappa, Vidushi Kapoor, Kodaganur S. Gopinath, D.C. Doval, K.S. Sabitha, Gaspar Taroncher-Oldenburg, Biswanath Majumder, Pradip Majumder, Mohammad Kohandel, and Aaron Goldman

N	50	
Age in years, median (range)	54 (21-80)	
Gender		
Male	20	40%
Female	29	58%
Not Reported	1	2%
Tumor Stage		
T4N2M0	37	74%
T3N0M0	1	2%
T3N1M0	1	2%
T3N2M0	1	2%
T4N1M0	3	6%
Not Reported	7	14%
Tumor Site		
Palate	2	4%
Maxilla	2	4%
Tongue	5	10%
Lower alveolus	10	20%
Buccal Mucosa	25	50%
Others	6	12%

Figure S1: Patient demographics head and neck squamous cell carcinoma (N=50). Related to Figure 1.

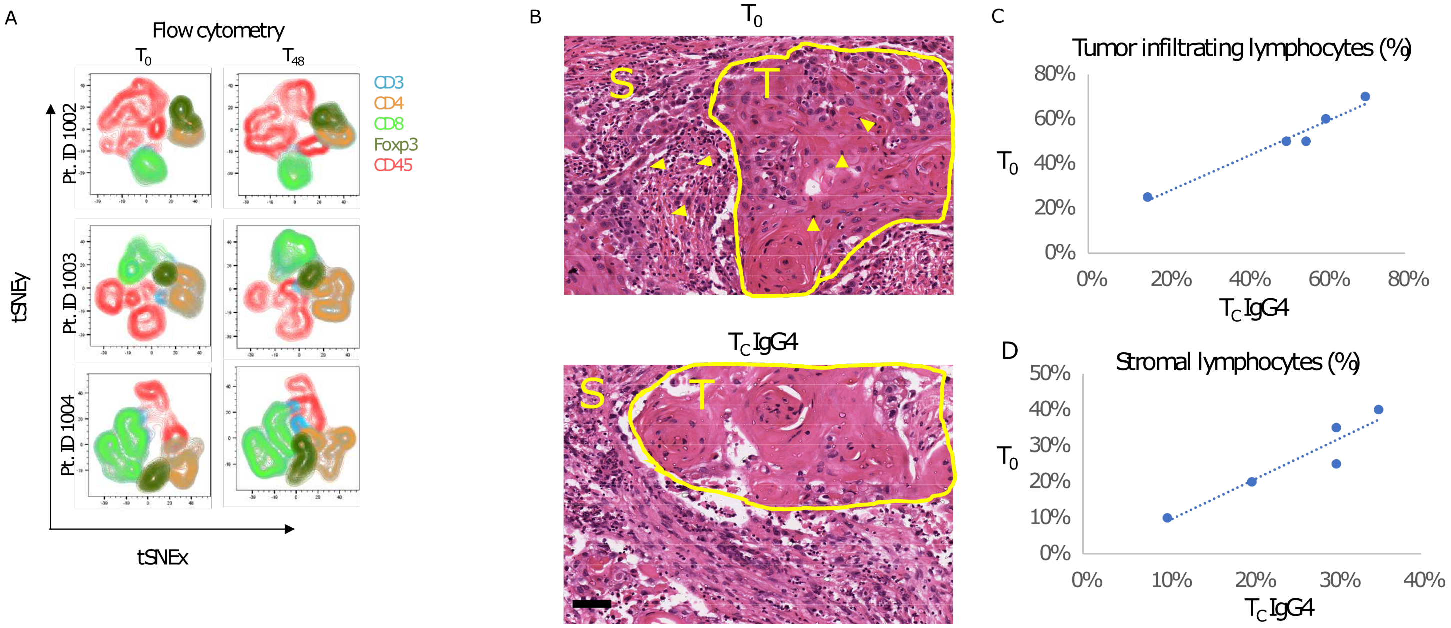

Figure S2: Profiling spatio-temporal immune fidelity comparing T_0 with unstimulated vehicle control (IgG4). Related to Figure 1. A. tSNE visualization of the tumor infiltrated immune population in baseline (T_0) and the ex-vivo vehicle control (IgG4) culture (48h) for 3 patient samples. The data is generated from flow cytometry of 3000 merged events for each sample with further sub-gating of the main T-cell populations. B. Representative H&E from HNSCC tumors at 200X magnification. Yellow line demarcates tumor (T) from stroma (S). Arrowheads indicate lymphocytes, shown as examples. Scale bar = 40mm. C-D. Blinded quantification by a clinical pathologist was performed to determine the percent (%) of tumor infiltrated lymphocytes (TIL) (B) or stromal lymphocytes (C). Linear regression and correlation of TIL and stromal lymphocytes in a pair-wise fashion of T_0 (baseline), and the patient-matched T_C Vehicle.

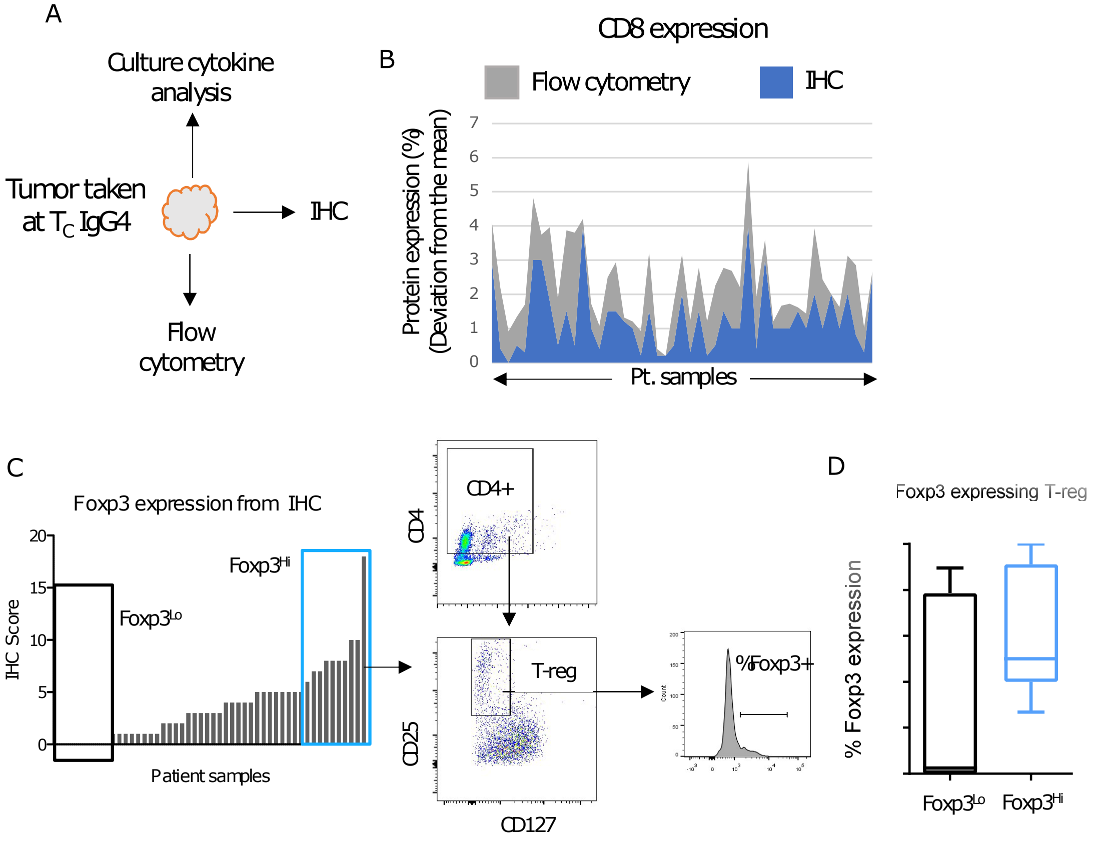

Figure S3: Testing preservation of immuno-biology by cross-analyzing multiple assays after culture, ex vivo. Related to Figure 2. A. Schematic shows the different molecular and biological assays that are employed to study tumor phenotype and culture media cytokines. B. Graph overlays the % CD8 protein expression determined by flow cytometry and immunohistochemistry (IHC). Data plotted as deviation from the mean of the respective analysis (IHC or flow), N=50 patient samples on x-axis. C. Histogram shows distribution of Foxp3 expression from the vehicle-treated cohort of 50 patient samples as determined by blinded quantification of IHC staining intensity by clinical pathology. Boxes indicate the highest and lowest Foxp3 expressing patient samples ($Foxp3^{Hi}$ and ^{Lo}). D. Histogram quantifies the % Foxp3 expression from T-reg cells determined by flow cytometry in the grouped patient samples $Foxp3^{Hi}$ and $Foxp3^{Lo}$ (Fig. Panel C).

Transparent methods

1 Systems biology model

Given the experimental measurements of cytokine expression and relative T-cell populations in ex-vivo human tumor cultures, and taking into account well-established immune cell interactions in the literature, we developed the interaction network depicted in Figure 4 of the manuscript.

An explanation of each of the cellular and protein species appearing in Figure 4 is presented in Table S1, along with its variable representation in the mathematical equations.

Table S1: Cellular and protein species in the interaction network in Figure 4.

**Cellular species**	**Description**	**Mathematical representation**
CD4+ Th0	Naive helper (CD4+) T-cell population	T_{N4}
CD4+ Th1	Type 1 helper T-cell population	Th_1
CD4+ Th2	Type 2 helper T-cell population	Th_2
Naive CD8+	Naive cytotoxic (CD8+) T-cell population	T_{N8}
CD8+ Tc	Cytotoxic (CD8+) T-cell population	T_c
DC	Dendritic cell population	–
Cancer	Cancer cell population	C
**Protein species**	**Description**	**Mathematical representation**
IL-4	Concentration of interleukin 4	$[IL\text{-}4]$
IL-6	Concentration of interleukin 6	$[IL\text{-}6]$
IL-12	Concentration of interleukin 12	$[IL\text{-}12]$
IFNγ	Concentration of interferon gamma	$[IFN\gamma]$
PD-1	Concentration of programmed cell death protein 1	$[PD\text{-}1]$
PD-L1	Concentration of programmed death-ligand 1	$[PD\text{-}L1]$
PD-1:PD-L1	Concentration of PD-1:PD-L1 protein complex	$[PD\text{-}1{:}PD\text{-}L1]$
**Drug species**	**Description**	**Mathematical representation**
Nivolumab	Concentration of the PD-1 inhibitor Nivolumab	$[A]$
–	Concentration of Nivolumab:PD-1 complex	$[A : PD\text{-}1]$

Below we explain each interaction in the network, along with its mathematical formulation, in addition to the key assumptions in the model. For each cellular species, it is assumed that cells proliferate via mitosis and die at rates that are proportional to their population size.

1. Time evolution of naive helper (CD4+) T-cell population:

$$\frac{dT_{N4}}{dt} = n_4 T_{N4} - \left(d_{1\text{-}12} T_{N4} \frac{[IL\text{-}12]}{q_{dIL12} + [IL\text{-}12]} + d_{1\text{-}IFN} T_{N4} \frac{[IFN\gamma]}{q_{IFN\text{-}1} + [IFN\gamma]} \right) \left(\frac{s_1}{s_1 + [PD\text{-}1 : PD\text{-}L1]} \right)$$
$$- \left(d_2 T_{N4} \frac{[IL\text{-}4]}{q_{dIL4} + [IL\text{-}4]} \right) \left(\frac{s_2}{s_2 + [PD\text{-}1 : PD\text{-}L1]} \right) \quad (1)$$

The first term describes the net proliferation of T_{N4} cells. The next two terms describe the differentiation of T_{N4} cells into Th_1 cells in the presence of IL-12 (Yates et al., 2000) (term 2) and IFNγ (Diehl and Rincón, 2002) (term 3). Both of these differentiation processes are inhibited by the PD-1:PD-L1 complex (Freeman et al., 2006; Okazaki and Honjo, 2006; Sznol and Chen, 2013). The last term describes the differentiation of T_{N4} cells into Th_2 cells in the presence of IL-4 (Diehl and Rincón, 2002; Yates et al., 2000), which is inhibited by the PD-1:PD-L1 complex (Freeman et al., 2006; Okazaki and Honjo, 2006; Sznol and Chen, 2013).

2. Time evolution of type 1 helper T-cell population:

$$\frac{dTh_1}{dt} = n_1 Th_1 + \left(d_{1\text{-}12} T_{N4} \frac{[IL\text{-}12]}{q_{dIL12} + [IL\text{-}12]} + d_{1\text{-}IFN} T_{N4} \frac{[IFN\gamma]}{q_{IFN\text{-}1} + [IFN\gamma]} \right) \left(\frac{s_1}{s_1 + [PD\text{-}1 : PD\text{-}L1]} \right) \tag{2}$$

The first term describes the net proliferation of Th_1 cells. The remaining terms describe the increase in the Th_1 cell population due to the differentiation of T_{N4} cells into Th_1 cells in the presence of IL-12 (Yates et al., 2000) (term 2) and IFNγ (Diehl and Rincón, 2002) (term 3), which is inhibited by the PD-1:PD-L1 complex (Freeman et al., 2006; Okazaki and Honjo, 2006; Sznol and Chen, 2013).

3. Time evolution of type 2 helper T-cell population:

$$\begin{aligned} \frac{dTh_2}{dt} = & \left(g_2 Th_2 + g_{2\text{-}4} Th_2 \frac{[IL\text{-}4]}{q_{gIL4} + [IL\text{-}4]} \right) \left(\frac{r_{IFN}}{r_{IFN} + [IFN\gamma]} \right) \\ & + \left(d_2 T_{N4} \frac{[IL\text{-}4]}{q_{dIL4} + [IL\text{-}4]} \right) \left(\frac{s_2}{s_2 + [PD\text{-}1 : PD\text{-}L1]} \right) - \delta_2 Th_2 \end{aligned} \tag{3}$$

The first term describes the proliferation of Th_2 cells due to mitosis, which is upregulated by IL-4 (Diehl and Rincón, 2002; Yates et al., 2000) (term 2). However, this cell proliferation is inhibited by IFNγ (Fishman and Perelson, 1994). The next term describes the increase in Th_2 population resulting from the differentiation of T_{N4} cells into Th_2 cells in the presence of IL-4 (Diehl and Rincón, 2002; Yates et al., 2000) (term 3), which is inhibited by the PD-1:PD-L1 (Freeman et al., 2006; Okazaki and Honjo, 2006; Sznol and Chen, 2013) complex. The last term describes natural death of the Th_2 cells.

4. Time evolution of naive cytotoxic (CD8+) T-cell population:

$$\frac{dT_{N8}}{dt} = n_8 T_{N8} - d_c T_{N8} \left(\frac{Th_1}{q_1 + Th_1} \right) \left(\frac{s_C}{s_C + [PD\text{-}1 : PD\text{-}L1]} \right) \tag{4}$$

The first term describes the net proliferation of T_{N8} cells. The second term describes the differentiation of T_{N8} cells into T_c cells in the presence of Th_1 (Ridge et al., 1998; Sakaguchi, 2000), which is inhibited by the PD-1:PD-L1 complex (Freeman et al., 2006; Okazaki and Honjo, 2006; Sznol and Chen, 2013).

5. Time evolution of cytotoxic (CD8+) T-cell population:

$$\frac{dT_c}{dt} = n_c T_c + g_{c-12} T_c \frac{[IL-12]}{q_{gIL12} + [IL-12]} + d_c T_{N8} \left(\frac{Th_1}{q_1 + Th_1} \right) \left(\frac{s_c}{s_c + [PD\text{-}1 : PD\text{-}L1]} \right) \tag{5}$$

The first term describes the net proliferation of T_c cells, which is upregulated by IL-12 (Lasek et al., 2014) (term 2). The third term describes the increase in the T_c cell population due to the differentiation of T_{N8} cells into T_c cells in the presence of DCs that have been activated by Th_1 (Ridge et al., 1998; Sakaguchi, 2000). This differentiation process is inhibited by the PD-1:PD-L1 complex (Freeman et al., 2006; Okazaki and Honjo, 2006; Sznol and Chen, 2013).

6. Time evolution of cancer cell population:

$$\frac{dC}{dt} = n_{Can} C - k_c C T_c \tag{6}$$

The first term describes the net proliferation of cancer cells and the second term describes the killing of cancer cells by T_c cells through mechanisms such as granzyme/perforin-induced apoptosis (Freeman et al., 2006; Trapani and Smyth, 2002).

7. Time evolution of IFN-γ concentration:

$$\frac{d[IFN\gamma]}{dt} = p_{1\text{-}IFN} Th_1 \left(\frac{r_{IL4}}{r_{IL4} + [IL\text{-}4]}\right)\left(\frac{r_{IL6}}{r_{IL6} + [IL\text{-}6]}\right) + p_{c\text{-}IFN} T_c - \delta_{IFN}[IFN\gamma] \tag{7}$$

Term one describes the secretion of IFNγ by Th_1 cells (Diehl and Rincón, 2002; Fishman and Perelson, 1994; Yates et al., 2000), which is inhibited by IL-4 (Fishman and Perelson, 1999) and IL-6 (Diehl and Rincón, 2002). The second term describes the secretion of IFNγ by T_c cells (Freeman et al., 2006), and the third term describes the natural decay of IFNγ.

8. Time evolution of IL-4 concentration:

$$\frac{d[IL\text{-}4]}{dt} = p_{2\text{-}4} Th_2 + p_{2\text{-}4\text{-}6} Th_2 \left(\frac{[IL\text{-}6]}{q_{IL6} + [IL\text{-}6]}\right) - \delta_{IL4}[IL\text{-}4] \tag{8}$$

The first term describes the secretion of IL-4 by Th_2 cells (Diehl and Rincón, 2002; Fishman and Perelson, 1994; Yates et al., 2000). The second term describes the additional secretion of IL-4 by Th_2 cells in the presence of IL-6 (Diehl and Rincón, 2002; Romagnani, 1997). The third term describes the natural decay of IL-4.

9. Time evolution of IL-6 concentration:

$$\frac{d[IL\text{-}6]}{dt} = p_{2\text{-}6} Th_2 + p_{Can\text{-}6} C - \delta_{IL6}[IL\text{-}6] \tag{9}$$

Term one describes the secretion of IL-6 by Th_2 cells (Fishman and Perelson, 1994). Antigen presenting cells produce IL-6 (Diehl and Rincón, 2002; Rincón et al., 1997) and we assume that the number of antigen presenting cells is directly proportional to the number of cancer cells (term 2). The third term describes the natural decay of IL-6.

10. Time evolution of IL-12 concentration:

$$\frac{d[IL\text{-}12]}{dt} = p_{Can\text{-}12} C + p_{1\text{-}12} Th_1 - \delta_{IL12}[IL\text{-}12] \tag{10}$$

Term one describes the production of IL-12 by DCs, which we assume to be directly proportional to the number of cancer cells (Rincón et al., 1997). Term two describes the additional production of IL-12 by DCs that are activated by Th_1 cells (Macatonia et al., 1995). The third term describes the natural decay of IL-12.

11. PD-1 concentration and its time evolution:

$$[PD\text{-}1] = \rho\,(Th_1 + Th_2 + T_c) \tag{11}$$

$$\frac{d[PD\text{-}1]}{dt} = \rho\left(\frac{dTh_1}{dt} + \frac{dTh_2}{dt} + \frac{dT_c}{dt}\right) - \beta_+[PD\text{-}1][PD\text{-}L1] + \beta_-[PD\text{-}1 : PD\text{-}L1] - \alpha_+[PD\text{-}1][A] + \alpha_-[A : PD\text{-}1] \tag{12}$$

PD-1 is expressed on all activated T-cells, i.e. Th_1, Th_2, and T_c (Freeman et al., 2006; Sznol and Chen, 2013), thus the total concentration of PD-1 is proportional to the sum of the T-cell populations, as indicated in Equation (11). We make the simplifying assumption that the same amount of PD-1 is expressed on all types of T-cells, thus the proportionality constants for each population are the same.

The time evolution of PD-1 is described by Equation (12). The first three terms describe the change in the PD-1 levels due to changing T-cell populations. The fourth term describes the binding of PD-1 to PD-L1 to form the PD-1:PD-L1 complex and the fifth term describes the dissociation

of the PD-1:PD-L1 complex (Freeman et al., 2006). The last two terms describe, respectively, the binding of PD-1 to Nivolumab and the dissociation of the Nivolumab:PD-1 complex (Sznol and Chen, 2013).

12. PD-L1 concentration and its time evolution:

$$[PD\text{-}L1] = \lambda\left(Th_1 + Th_2 + T_c + C\right) + \lambda_{Can\text{-}IFN} C \left(\frac{[IFN\gamma]}{q_{IFN\text{-}PDL1} + [IFN\gamma]}\right) \tag{13}$$

$$\begin{aligned}\frac{d[PD\text{-}L1]}{dt} &= \lambda\left(\frac{dTh_1}{dt} + \frac{dTh_2}{dt} + \frac{dT_c}{dt} + \frac{dC}{dt}\right)\\ &\quad + \lambda_{Can\text{-}IFN}\frac{dC}{dt}\left(\frac{[IFN\gamma]}{q_{IFN\text{-}PDL1} + [IFN\gamma]}\right)\\ &\quad - \beta_+[PD\text{-}1][PD\text{-}L1] + \beta_-[PD\text{-}1 : PD\text{-}L1]\end{aligned} \tag{14}$$

PD-L1 is expressed on all activated T cells, i.e. Th_1, Th_2, T_c (Freeman et al., 2006; Okazaki and Honjo, 2006) as well as cancer cells (Freeman et al., 2006; Okazaki and Honjo, 2006; Sznol and Chen, 2013), thus the total concentration of PD-L1 is in part proportional to the sum of the T-cell and cancer cell populations, as indicated by the first four terms in Equation (13). We make the simplifying assumption that the PD-L1 expression is identical for all types of cells. In addition, the expression of PD-L1 by cancer cells is upregulated by IFNγ (Freeman et al., 2006; Okazaki and Honjo, 2006; Sznol and Chen, 2013), as indicated by the fifth term in Equation (13).

The time evolution of PD-L1 is described by Equation (14). The first five terms describe the change in PD-L1 levels due to changing T-cell and cancer cell populations. We make the simplifying assumption that the proteins reach their steady state values instantaneously with respect to the time scale of the changes in cell populations (i.e. the cell division rate) so that $\frac{d[IFN\gamma]}{dt} \approx 0$. The last two terms describe the binding of PD-1 to PD-L1 and the dissociation of the PD-1:PD-L1 complex (Freeman et al., 2006), respectively.

13. Time evolution of PD-1:PD-L1 complex concentration:

$$\frac{d[PD\text{-}1 : PD\text{-}L1]}{dt} = \beta_+[PD\text{-}1][PD\text{-}L1] - \beta_-[PD\text{-}1 : PD\text{-}L1] \tag{15}$$

The first term describes the binding of PD-1 to PD-L1 and the second term describes the dissociation of the PD-1:PD-L1 complex (Freeman et al., 2006).

14. Time evolution of free Nivolumab concentration:

$$\frac{d[A]}{dt} = \tilde{A}(t) - \alpha_+[A][PD\text{-}1] + \alpha_-[A : PD\text{-}1] - \delta_A[A] \tag{16}$$

The first term describes the introduction of Nivolumab into the system, which may be time-dependent, depending on the treatment schedule. The second term describes the binding of PD-1 to Nivolumab, resulting in the formation of the Nivolumab:PD-1 complex, and the third term describes the dissociation of the Nivolumab:PD-1 complex (Sznol and Chen, 2013). We make the assumption that the dissociation constant $K_\alpha \equiv \alpha_-/\alpha_+ \ll K_\beta \equiv \beta_-/\beta_+$ so that Nivolumab has a higher binding affinity for PD-1 than PD-L1 does, which allows the drug to displace PD-L1 from the PD-1:PD-L1 complex. Further, in simulations we assume that the rate of association of PD-1 is equivalent for Nivolumab and PD-L1, i.e. $\alpha_+ = \beta_+$, which removes a kinetic parameter from the system. The fourth term in the equation describes the natural decay of Nivolumab.

15. Time evolution of Nivolumab:PD-1 complex concentration:

$$\frac{d[A:PD\text{-}1]}{dt} = \alpha_+[A][PD\text{-}1] - \alpha_-[A:PD\text{-}1] \tag{17}$$

The first term describes the binding of Nivolumab with PD-1 to form the Nivolumab:PD-1 complex, and the second term describes the dissociation of the drug complex (Sznol and Chen, 2013).

In Table S2 we give a description of the kinetic parameters that appear in the above equations in the mathematical model.

Table S2: Description of the kinetic parameters in the systes biology model. Related to the interaction network in Figure 4.

Number	Name	Description
1	n_4	Net proliferation rate of T_{N4} cells
2	n_8	Net proliferation rate of T_{N8} cells
3	n_1	Net proliferation rate of Th_1 cells
4	n_c	IL12-independent net proliferation rate of T_c cells
5	n_{Can}	Net proliferation rate of cancer cells
6	g_2	IL4-independent growth rate of Th_2 cells
7	$g_{2\text{-}4}$	IL4-dependent growth rate of Th_2 cells
8	$g_{c\text{-}12}$	IL12-dependent growth rate of T_c cells
9	δ_2	Death rate of Th_2 cells
10	$d_{1\text{-}IFN}$	IFNγ-dependent differentiation rate of T_{N4} cells into Th_1 cells
11	$d_{1\text{-}12}$	IL12-dependent differentiation rate of T_{N4} cells into Th_1 cells
12	d_2	IL4-dependent differentiation rate of T_{N4} cells into Th_2 cells
13	d_c	Rate of differentiation of T_{N8} cells into T_c cells
14	k_c	Rate of cancer cell killing by T_c cells
15	$p_{1\text{-}IFN}$	Rate of production of IFNγ by Th_1 cells
16	$p_{2\text{-}4\text{-}6}$	IL6-dependent production of IL-4 by Th_2 cells
17	p_{Can-6}	Rate of production of IL-6 by antigen presenting cells (assumed proportional to the number of cancer cells)
18	p_{Can-12}	Rate of production of IL-12 by DCs (assumed proportional to the number of cancer cells)
19	δ_{IFN}	Decay rate of IFNγ
20	δ_{IL4}	Decay rate of IL-4
21	δ_{IL6}	Decay rate of IL-6
22	δ_{IL12}	Decay rate of IL-12
23	δ_A	Decay rate of Nivolumab
24	q_1	Half-maximal Th_1 cell population for T_{N8} differentiation into T_c cells

25	$q_{IFN\text{-}1}$	Half-maximal IFNγ concentration for IFNγ-dependent differentiation of T_{N4} cells into Th_1 cells
26	$q_{IFN\text{-}PDL1}$	Half-maximal IFNγ concentration for IFNγ-dependent PD-L1 expression by cancer cells
27	q_{gIL4}	Half-maximal IL-4 concentration for IL4-dependent proliferation of Th_2 cells
28	q_{dIL4}	Half-maximal IL-4 concentration for IL4-dependent differentiation of T_{N4} cells into Th_2 cells
29	q_{IL6}	Half-maximal IL-6 concentration for IL6-dependent production of IL-4 by Th_2 cells
30	q_{dIL12}	Half-maximal IL-12 concentration for IL12-dependent differentiation of T_{N4} cells into Th_1 cells
31	q_{gIL12}	Half-maximal IL-12 concentration for IL12-dependent proliferation of T_c cells
32	r_{IFN}	Half-maximal IFNγ concentration for IFNγ-dependent inhibition of Th_2 proliferation
33	r_{IL4}	Half-maximal IL-4 concentration for IL4-dependent inhibition of IFNγ production by Th_1 cells
34	r_{IL6}	Half-maximal IL-6 concentration for IL6-dependent inhibition of IFNγ production by Th_1 cells
35	ρ	Per-cell expression level of PD-1
36	λ	Per-cell expression level of PD-L1
37	$\lambda_{Can\text{-}IFN}$	IFNγ-dependent PD-L1 expression per cancer cell
38	β_+	Rate of association of PD-1 and PD-L1
39	β_-	Rate of dissociation of PD-1:PD-L1 complex
40	α_-	Rate of dissociation of Nivolumab:PD-1 complex
41	s_1	Half-maximal PD-1:PD-L1 concentration for inhibition of T_{N4} differentiation into Th_1 cells
42	s_2	Half-maximal PD-1:PD-L1 concentration for inhibition of T_{N4} differentiation into Th_2 cells
43	s_c	Half-maximal PD-1:PD-L1 concentration for inhibition of T_{N8} differentiation into T_c cells
44	$p_{1\text{-}12}$	Rate of IL-12 production by Th_1 cells
45	$p_{2\text{-}4}$	Rate of IL6-independent production of IL-4 by Th_2 cells
46	$p_{2\text{-}6}$	Rate of IL-6 production by Th_2 cells
47	$p_{c\text{-}IFN}$	Rate of IFNγ production by T_c cells

2 Systems biology parameter values

In Table S3, we present the numerical values of the parameters and initial conditions (protein levels and relative T-cell populations) that match to the average patient data (see “nominal value” column), as well as the corresponding units (see “units” column). We use the abbreviation “min” to denote a timescale of minutes. We also present a range for each parameter which was used for searching the parameter space with Matlab’s genetic algorithm to match the average patient data as well as for performing the

global sensitivity analysis (see "range" column). The ranges presented for the protein levels and T-cell fractions that were obtained from the patient data are set by the minimum and maximum experimentally measured values for all patients, and were used for the global sensitivity analysis. When using the genetic algorithm to match the average patient data, the T-cell fractions were set to the average of all patients without treatment, and the protein levels were sampled from a range set by the average +/- one standard deviation, as explained in the main text.

We note that parameters 44-47 do not have a specified range since they were calculated at the beginning of each simulation by assuming the initial protein levels are steady state protein levels (Equations 7-10) using the initial T-cell population values, and additionally imposing the constraint that all parameters are non-negative. Thus with the local and global sensitivity analysis, it was necessary to re-calculate parameters 44-47 for each simulation.

To ensure that Nivolumab has a higher binding affinity for PD-1 than PD-L1 does, we also imposed the constraint (parameter 40 $<$ 0.1 parameter 39) for each simulation.

Additionally, we note that the PD-1 and PD-L1 concentrations were initialized for each simulation using Equations 11 and 13, respectively, with the initial cell populations and relevant protein level. Finally we point out that in an initial analysis, we used a larger upper bound for the net proliferation rate of cancer cells, parameter 5. In some cases, this led to nonphysical growth of the cancer population over the three day treatment window when there was no treatment response. In these cases, the model output was most sensitive to parameters controlling the CD8+ cytotoxic T-cell population and its efficiency at killing the cancer cells. We present additional important notes below the table.

Table S3: Values and ranges of the kinetic parameters, initial protein levels, and initial T-cell populations used for local and global sensitivity analysis. Related to Figures 5 and 6.

Parameter	Nominal value	Range	Units	Reference
1	2.5×10^{-1}	$\ln(2)/20 - \ln(2)$	day^{-1}	estimated from (Fishman and Perelson, 1994) (Fishman and Perelson, 1999)
2	3.5×10^{-2}	$\ln(2)/20 - \ln(2)$	day^{-1}	estimated from (Fishman and Perelson, 1994) (Fishman and Perelson, 1999)
3	4.8×10^{-2}	$\ln(2)/20 - \ln(2)$	day^{-1}	estimated from (Fishman and Perelson, 1994) (Fishman and Perelson, 1999)
4	4.1×10^{-2}	$\ln(2)/20 - \ln(2)$	day^{-1}	estimated from (Fishman and Perelson, 1994) (Fishman and Perelson, 1999)
5	7.0×10^{-3}	$\ln(2)/100 - \ln(2)/10$	day^{-1}	estimated
6	3.8×10^{-2}	$\ln(2)/20 - \ln(2)$	day^{-1}	estimated from (Fishman and Perelson, 1994) (Fishman and Perelson, 1999)
7	3.5×10^{-2}	$\ln(2)/20 - \ln(2)$	day^{-1}	estimated from (Fishman and Perelson, 1994) (Fishman and Perelson, 1999)
8	3.6×10^{-2}	$\ln(2)/20 - \ln(2)$	day^{-1}	estimated from (Fishman and Perelson, 1994) (Fishman and Perelson, 1999)

9	1.2×10^{-2}	$\ln(2)/60 - \ln(2)/7$	day^{-1}	estimated from (Fishman and Perelson, 1994) (Fishman and Perelson, 1999)
10	1.9×10^{-1}	$\ln(2)/20 - \ln(2)$	day^{-1}	estimated from (Morel et al., 1992)
11	3.6×10^{-2}	$\ln(2)/20 - \ln(2)$	day^{-1}	estimated from (Morel et al., 1992)
12	2.1×10^{-2}	$\ln(2)/20 - \ln(2)$	day^{-1}	estimated from (Morel et al., 1992)
13	2.3×10^{-2}	$\ln(2)/20 - \ln(2)$	day^{-1}	estimated from (Morel et al., 1992)
14	1.1×10^{-5}	$10^{-5} - 10^{-1}$	T_c cell$^{-1}\cdot$ day^{-1}	estimated
15	1.3×10^{-3}	$6.5 \times 10^{-4} - 1.7 \times 10^{-2}$	$\frac{\text{pg/mL}}{Th_1 \text{ cell} \cdot \text{day}}$	estimated
16	1.6×10^{-4}	$1.4 \times 10^{-7} - 1.4\times^{-2}$	$\frac{\text{pg/mL}}{Th_2 \text{ cell} \cdot \text{day}}$	estimated
17	1.8×10^{-2}	$7.2 \times 10^{-3} - 7.2 \times 10^{-1}$	$\frac{\text{pg/mL}}{\text{cancer cell} \cdot \text{day}}$	estimated
18	1.2×10^{-3}	$1.2 \times 10^{-3} - 1.4 \times 10^{-2}$	$\frac{\text{pg/mL}}{\text{cancer cell} \cdot \text{day}}$	estimated from (Lai and Friedman, 2017)
19	$\ln(2)/1000$	$\ln(2)/1000 - \ln(2)/60$	min^{-1}	estimated from (Fishman and Perelson, 1999)
20	7.0×10^{-4}	$\ln(2)/1000 - \ln(2)/60$	min^{-1}	estimated from (Fishman and Perelson, 1999)
21	$\ln(2)/1000$	$\ln(2)/1000 - \ln(2)/60$	min^{-1}	estimated from (Fishman and Perelson, 1999)
22	4.8×10^{-4}	$\ln(2)/1440 - \ln(2)/600$	min^{-1}	estimated from (Lai and Friedman, 2017)
23	4.8×10^{-2}	$\ln(2)/15 - \ln(2)/10$	day^{-1}	estimated from (Lai and Friedman, 2017)
24	1.6×10^{2}	$1 - 10^{5}$	Th_1 cells	estimated
25	4.0×10^{-1}	$10^{-3} - 10^{2}$	$[IFN\gamma]$ (pg/mL)	estimated
26	6.3×10^{-1}	$10^{-3} - 10^{2}$	$[IFN\gamma]$ (pg/mL)	estimated
27	4.03	$10^{-3} - 10^{3}$	$[IL\text{-}4]$ (pg/mL)	estimated
28	8.4×10^{-1}	$10^{-3} - 10^{3}$	$[IL\text{-}4]$ (pg/mL)	estimated
29	1.3×10^{2}	$10^{2} - 10^{4}$	$[IL\text{-}6]$ (pg/mL)	estimated
30	6.3×10^{-3}	$10^{-3} - 10^{2}$	$[IL\text{-}12]$ (pg/mL)	estimated
31	3.4×10^{-2}	$10^{-3} - 10^{2}$	$[IL\text{-}12]$ (pg/mL)	estimated
32	8.9×10^{-2}	$10^{-3} - 10^{2}$	$[IFN\gamma]$ (pg/mL)	estimated
33	7.5×10^{-1}	$10^{-1} - 10^{3}$	$[IL\text{-}4]$ (pg/mL)	estimated
34	1.4×10^{2}	$10^{2} - 10^{4}$	$[IL\text{-}6]$ (pg/mL)	estimated
35	9.9	$10^{-6} - 10^{1}$	(pg/mL)/T-cell	estimated from (Lai and Friedman, 2017)

36	1.0×10^{1}	$10^{-6} - 10^{1}$	(pg/mL)/cell	estimated from (Lai and Friedman, 2017)
37	1.8×10^{-4}	$10^{-10} - 10^{-1}$	(pg/mL)/cancer cell	estimated from (Lai and Friedman, 2017)
38	1.7×10^{-3}	$1.4 \times 10^{-4} - 1.4 \times 10^{-1}$	$\left((\text{pg/mL}) \cdot \text{day}\right)^{-1}$	estimated
39	1.5	$1.4 - 1.4 \times 10^{2}$	day^{-1}	estimated
40	3.5×10^{-3}	$1.4 \times 10^{-3} - 1.4 \times 10^{-1}$	$\left((\text{pg/mL}) \cdot \text{day}\right)^{-1}$	estimated
41	4.9×10^{1}	$10^{-3} - 10^{5}$	$[PD\text{-}1 : PD\text{-}L1]$ (pg/mL)	estimated
42	2.1	$10^{-3} - 10^{5}$	$[PD\text{-}1 : PD\text{-}L1]$ (pg/mL)	estimated
43	1.9×10^{1}	$10^{-3} - 10^{5}$	$[PD\text{-}1 : PD\text{-}L1]$ (pg/mL)	estimated
44	7.7×10^{-5}	see text	$\frac{\text{pg/mL}}{Th_2 \text{ cell} \cdot \text{day}}$	–
45	3.7×10^{-6}	see text	$\frac{\text{pg/mL}}{Th_2 \text{ cell} \cdot \text{day}}$	–
46	1.1×10^{-1}	see text	$\frac{\text{pg/mL}}{T_c \text{ cell} \cdot \text{day}}$	–
47	3.0×10^{-8}	see text	$\frac{\text{pg/mL}}{T_c \text{ cell} \cdot \text{day}}$	–
**Protein**	**Nominal value**	**Range**	**Units**	**Reference**
IFNγ	0.45	0.18 – 482.31	pg/mL	patient data
IL-12	1.54	1.82 – 11.44	pg/mL	patient data
IL-6	3339.16	149.15 – 35884.0	pg/mL	patient data
IL-4	0.11	0.10 – 61.37	pg/mL	patient data
**Cell fraction**	**Nominal value**	**Range**	**Units**	**Reference**
Cancer fraction	0.81	0.1 – 0.9*	–	estimated
TN8 fraction	0.65	0.21–0.97	–	patient data
Tc fraction	0.10	0.0–0.59	–	patient data
CD4+ fraction	0.25	0.01–0.69**	–	patient data
Th1 fraction	3.9×10^{-4}	0 – 0.99	–	estimated
Th2 fraction	8.6×10^{-4}	0 – 0.99***	–	estimated

*The tumor is assumed to consist of a population of cancer cells and a population of immune cells. Thus with the nominal values given in Table 3, 81% of the tumor is cancer cells and the remaining 19% is the total immune cell population.
**The total immune cell population consists of naive CD8+ T-cells (T_{N8}), CD8+ cytotoxic T-cells (T_c), and a population of CD4+ cells, thus we always impose the constraint (TN8 fraction + Tc fraction + CD4+ fraction) = 1.
***The CD4+ fraction is further subdivided into naive helper CD4+ T-cells (T_{N4}), type 1 helper T-cells (Th_1) and type 2 helper T-cells (Th_2), thus we always impose the constraint (Th1 fraction + Th2 fraction + TN4 fraction) = 1.

3 Ex vivo Culture

HNSCC patients were recruited from multiple hospitals in India with approval from institutional review board (IRB) and institutional ethical committee (IEC). Patient-consented tumor biopsies or surgical tissues, in addition to blood specimens were transported to Mitra Biotech, Bangalore in a transport container (Crēdo CubeTM (Peli Bio Thermal, Plymouth MN) in transport buffer containing a defined tissue culture media recipe (described in detail below) and processed within 24-48 h post-excision. The time of excision was captured on surgical reports. Both blood and tissue samples were shipped in temperature-controlled containers, also containing temperature-loggers to maintain a temperature of 4-7°C. Details of patient demography collected are outlined in Supplemental Figure 1. Quality control (QC) of the sample include: (1) absence of blood hemolysis, (2) arrival of patient tumors and blood specimens at a temperature range of 4-7°C (3) a minimum tumor content, which is evaluated by a clinical pathologist on HE-stained FFPE tissue (T0 tissue), of 20%. Approximately 50% of samples that arrived at the lab were deemed suitable, by the quality control criteria described above, to be further processed in the ex vivo system. Tumor tissues were then moved from the transport chamber into warmed (37°C) tissue culture media (recipe described in detail below), dissected into uniform slices using manual fragmentation procedures. Tissue slices were maintained in customized tumor matrix protein (TMP) coated plates as described earlier (Brijwani et al., 2017; Majumder et al., 2015). Briefly, TMP were previously identified using a tandem liquid chromatography mass spectrometry (LC/MS) approach which elucidated the varying concentrations of each protein that are typically found within head and neck tumors derived from human patients. Sterile recombinant human TMP are then coated onto tissue culture plates and used for the tissue culture procedure, outlined in Figure 1A. Tissue fragments (approximately 300 μm - 2 mm in size) were then placed into each well of a 48-well plate coated in TMP and incubated with 500 μl of tissue culture media (RPMI containing 20% fetal bovine serum, 2% autologous patient serum, penicillin, streptomycin. 1 Insulin-Transferrin-Selenium (ITS, Life Technologies. 41400-045), 1 GlutaMAX (Life Technologies. 35050-061) and 1 penicillin, streptomycin and amphotericin B (Life Technologies. 15140-122). Drug (described below) was then incubated with each tissue fragment. Experiments were performed in replicate of a maximum of four individual tissue fragments receiving drug and a minimum of three for up to 72 hours. Tissue culture media and drug removed and replaced every 24 hours. Tissue culture supernatants were collected at 0 hours (baseline) & 48-72 hours post-culture in the presence of protease and phosphatase inhibitors and stored at -80°C until further analysis. Similarly, tumor slices were collected at 0 hour (baseline) & post-culture, placed in RNAlater (Ambion, Thermo-Fisher Scientific) and processed for subsequent analyses (described in detail in each section below).

4 Drugs

The anti-PD-1 antibody, nivolumab (Opdivo, Bristol Myers Squibb) was dissolved into a 1x phosphate buffered saline (PBS) at pH7.2 and stored in aliquots at -80°C for one-time use (i.e. not repeated freeze/thawed). Isotype control antibody (Ultra-LEAF^{TM} Purified Human IgG4 Isotype Control, Biolegend catalog # 403702) was used to compare as a vehicle control or negative control against the test antibody, i.e. nivolumab. IgG4 control and nivolumab were used at the concentration of 132 μg/ml. This dose was selected based on the published clinical cmax area under the curve (Brahmer et al., 2010).

5 Multiplex Cytokine Analysis

The tissue culture supernatants (25 μl) were used to measure the secreted profile of cytokine analytes, incubated with 25 μl of beads for 1 h and 25 μl biotinylated detection antibody for 30 min. The complex

was spiked with 25 μl of Streptavidin-PE and analyzed for cytokine profiling using Luminex200 (Luminex, USA) platform. The cell-free supernatant (25 μl) was run on one or multiple Millipore Milliplex plates, customized for the analytes selected. For each plate, a set of standard curves was run to ensure accurate evaluation of the concentration of each analyte and the integrity of the assay. Each plate was read on the Luminex 200. Concentrations of each analyte was interpolated from their respective standard curve using the Milliplex Analyst software (Millipore, USA). Data from multiple plates were compiled and analyte fold changes, relative to vehicle controls, was calculated using an appropriate graphing and statistical software.

6 Enzymatic Dissociation of Tumor Tissues

For flow cytometric analysis, tumor tissues post-culture ex vivo in the presence of vehicle (IgG4) or nivolumab were subject to single cell dissociation. Tissue slices were transferred into gentle MACS C tube in FBS free media containing enzyme mix (Enzyme H, Enzyme R, and Enzyme A) (Tumor Dissociation Kit, Miltenyi Biotec, USA). Tissue slices were dissociated using the h_tumor_01.01, h_tumor_02.01 and h_tumor_03.01 dissociation programs in gentle MACS Dissociator (Miltenyi Biotec). The enzyme mix was inactivated after incubation at 37°C for 30 min. The single cell suspension was passed through 70 μm strainer, washed and resuspended in FACS buffer (2% FBS in PBS) for subsequent staining.

7 Flow Cytometry

Following enzymatic dissociation, single cell suspensions from T0 baseline and T72 culture (72 hours post culture vehicle control) obtained from culture were stained with the following antibodies: anti-CD45 AF700 (clone 560566), anti-CD4 Pe-Cy7 (557852), anti CD8-APC-H7(641400), anti-CD14 PE-Cy5.5 (562692), anti-Foxp3 PE (560082), all from BD Bioscience, anti-CD3 BV510 (317332, Biolegend). The live cells were gated using Live-Dead Blue fixable cell stain method (L23105, Thermo Fisher Scientific). Flow cytometry acquisition was performed in BD LSR Fortessa. To assess the phenotypic modulation post treatment with the test arms, single cell suspensions obtained following enzymatic dissociation were divided in two parts: one part was stained with a CD8 panel and the other with a T-reg panel. The following antibodies were used for flow cytometry analysis: CD8 cocktail (anti- IFNγ FITC/ anti-CD69 PE/ anti-CD8 PerCP-Cy^{TM}5.5/ anti-CD3 APC, 346048, BD Bioscience), T-reg Cocktail (anti-CD4 FITC/ anti-CD25 PE-Cy7/ anti-CD127 Alexa Fluor 647,560249, BD Bioscience) and anti-Foxp3 PE (560082, BD Bioscience). All antibodies were used according to manufacturers instructions along with recommended buffers. Flow cytometry acquisition was performed in BD FACS Canto II (BD Bioscience). All data were analysed using FlowJo software. For tSNE plots for T0 baseline vs T72 comparison, cells were first gated on live and singlets, then down-sampled and concatenated prior to visualization with the built in tSNE module in FlowJo. The settings for computation were as follows: iterations = 1000; perplexity = 20, learning rate = 200, theta = 0.5.

8 Immunohistochemistry

Tissue sections were deparaffinized followed by rehydration and soaked in Antigen Unmasking Solution (Vector Labs) for 10 minutes followed by retrieval. Following protein blocking, FFPE tissue sections were incubated with appropriate primary antibodies (anti-Ki-67, Dako, envision kit, 1:400, and anti-caspase 3c (rabbit) from CST, 1:600 dilution). Validated positive and negative controls were included for every IHC assay. Each IHC result was evaluated by two independent experts and any differences in observation both experts came to a consensus as described previously (Bressenot et al.,

2009; Vaira et al., 2010). Antibodies used: anti-human CD8 antibody (rabbit polyclonal, Abcam, cat Ab4055,1:300 dilution), anti-human-PD-L1 antibody (rabbit monoclonal, Cell Signaling Technology, cat# 13684, clone E1L3N, 1:100 dilution) and anti-human FoxP3 antibody (mouse monoclonal, Abcam, UK, cat# ab22510, 1:100 dilution). A compatible secondary antibody (100 μl) was incubated for an optimized time period in humidified condition (Signal stain(R) Boost IHC detection reagent HRP Rabbit, Cell Signaling Technology- 8114s or Signal stain(R) Boost IHC detection reagent HRP Mouse Cell Signaling Technology- 8125s wherever applicable). Staining was visualized with freshly prepared DAB + Chromogen followed by DAB detection system (Vector Lab). Slides were counterstained in Harris hematoxylin (Merck-6092530121730), dehydrated through graded ethanol solutions, cleared in xylene and cover slipped. IHC slides were examined using a light microscope (DM2500, Leica, USA) and quantified by scoring the level of positivity and intensity on a scale of 0-100 by a clinical pathologist. All slides were examined independently by two experienced histopathologists in a blinded fashion. Representative images were captured in 200X magnification using Leicas inbuilt camera (DFC 450 C).

9 Bioinformatics

Published data used for analysis from either NanoString (Chen et al. (Chen et al., 2016)) or RNA Seq (Riaz et al. (Riaz et al., 2017)) were obtained from the published supplemental data or GEO accession: GSE91061, respectively. The Riaz et al data set was filtered for samples that did not have the exact pre/on treatment pair and for nave-immune checkpoint inhibitor treated patient samples, resulting in a total of 17 samples used for analysis in this study. The genes used to identify a Th1-related phenotype were compiled from various literature sources and include: CASP1, CCL3, CCL4, CCRI1, CCR2, CCR5, CD38, CLU, CD55, CSF2, CTLA4, CXCR3, GATA3, IFNG, NKFBIA, IL12RB2, LTA, PRF1, CCL5, SPP1, STAT1, STAT4, TBX21, TNF. The variance calculation for control and treatment groups was performed to detect the change in variance of a gene signature between samples after drug pressure. Data was transformed to log2 Z-scores to obtain mean of 0 and standard deviation of 1. To calculate the same in Cytokines, Flow Cytometry and IHC datasets a small value of 0.1 was added to the data before log2 transformation to prevent infinite values after log, keeping the rest of the analysis the same.

10 Statistical Analysis

1-sample Kolmogorov-Smirnov test was used to assess the data distribution normality. A non-parametric Mann-Whitney U test was used to determine statistical significance with a two-tailed p-value. Spearman rho was calculated to determine the correlation coefficient between paired samples in the data sets from Figure 1C. GraphPad Prism v 7.0 was used to perform statistical calculations.

11 Mathematical modeling and numerical simulations

The interactions between different immune cell populations, between immune cells and cytokines, and between immune cells and cancer cells that were included in the systems biology model are well established in the literature (see Section 1 for more details).

We briefly describe here the cell populations comprising the model and the main interactions. The model consists of a population of nave CD4+ helper T-cells (CD4+ Th0), type1 helper T-cells (CD4+ Th1), type 2 helper T-cells (CD4+ Th2), nave CD8+ cytotoxic T-cells (nave CD8+), CD8+ cytotoxic T-cells (CD8+ Tc), and a cancer cell population. Cell proliferation and natural death are assumed for all cell populations in the model. Additionally, the CD4+ Th0 cells can differentiate into either CD4+ Th1

cells or CD4+ Th2 cells. The first differentiation process is mediated by the cytokines IL-4 and IL-6. IL-12 and IFNγ mediate the second differentiation process. Nave CD8+ cells differentiate into CD8+ Tc cells in the presence of CD4+ Th1 cells, and the proliferation rate of CD8+ Tc cells is increased by IL-12 expression level. CD8+ Tc cells kill the cancer cells. All activated T-cells (CD4+ Th1, CD4+ Th2, CD8+ Tc) express PD-1 and PD-L1. Cancer cells also express PD-L1, which is mediated by IFNγ expression. PD-1 and PD-L1 form a protein complex which inhibits all T-cell differentiation processes. The production of cytokines depends on the T-cell populations and cancer cell population, and there are several feedback loops which affect the production rates. We refer the reader to Section 1 for specific details.

To determine the values of the nominal parameters, we used the MATLAB genetic algorithm with the ode15s solver to integrate the system of coupled ODEs, while simulating the treatment protocol and forcing the simulated cytokine and T-cell populations to match to the average patient data. While performing the parameter search using MATLAB genetic algorithm, the parameter ranges were set to previously reported biologically relevant ranges when possible (see Section 1 for more details). To simulate the 72-hour treatment protocol on the model system we administered 132 μg/ml of nivolumab at t=0 h, t=24 h, and t=48 h. Drug washout between subsequent doses of nivolumab was simulated by setting the free drug level to zero immediately before administering the next dose. Over the treatment window, the cytokine expression levels were forced to lie between average +/- one standard deviation of the patient data (with nivolumab treatment) at t=24 h, 48 h, and 72 h, and the T-cell populations were forced to lie between average +/- one standard deviation at t=72 h (with nivolumab treatment). Using this approach, it is conceivable that there could be many sets of parameters that fit the average patient data. The nominal parameter set that we obtained is presented in Table 3 below.

To perform the local sensitivity analysis, each kinetic parameter was varied one-at-a-time by +1%, and the system was subsequently simulated with the perturbed parameter value. The relative sensitivity, S, was then calculated using the following equation,

$$S = \frac{(\tilde{C}_{72} - C_{72})/C_{72}}{(\tilde{p} - p)/p},$$

where p is the nominal parameter value, $\tilde{p}$ is the perturbed parameter value, C_{72} is the size of the cancer cell population after the 72-hour treatment protocol with the nominal parameter set, and $\tilde{C}_{72}$ is the size of the cancer cell population after the 72-hour treatment protocol with the perturbed parameter set. This process was repeated for all parameters in the model, as well as for the initial cytokine levels and initial T-cell populations.

To efficiently sample the parameter space (i.e. to generate the parameter sets and initial conditions) for the global sensitivity analysis, the Latin hypercube sampling method (McKay et al., 1979) was used. The ranges of the kinetic parameters, initial cytokine levels, and initial T-cell levels used for the Latin hypercube sampling are presented in Table 3 below; the ranges for initial cytokine levels and initial T-cell levels were set by the lowest and highest values expressed in the untreated patient data. In order to elucidate which patient features were necessary to capture the variability in patient response to treatment, the global sensitivity analysis was conducted in several steps. First, the kinetic parameters in the model were held fixed to the nominal values used for the local sensitivity analysis and the initial T-cell populations were fixed to the average values of the patient data (without treatment). Several thousand treatment simulations were then performed, where the initial cytokine levels were sampled within the range presented in Table 3 below. Next, we kept the kinetic parameters held fixed and varied all the initial cytokine levels and initial T-cell populations simultaneously. Specifically, using the Latin hypercube sampling method, 50,000 sets of initial cytokine levels and T-cell levels were generated within the ranges presented in Table 3, then the 72-hour treatment protocol was simulated for each set of initial conditions. Lastly, we allowed all the kinetic parameters and initial conditions to vary. Particularly, we generated 50,000 sets of parameters/initial conditions, which resulted in a large variability in the nature and the strength of the response to treatment.

References

J. R. Brahmer, C. G. Drake, I. Wollner, J. D. Powderly, J. Picus, W. H. Sharfman, E. Stankevich, A. Pons, T. M. Salay, T. L. McMiller, et al. Phase i study of single-agent anti–programmed death-1 (mdx-1106) in refractory solid tumors: safety, clinical activity, pharmacodynamics, and immunologic correlates. *Journal of clinical oncology*, 28(19):3167, 2010.

A. Bressenot, S. Marchal, L. Bezdetnaya, J. Garrier, F. Guillemin, and F. Plénat. Assessment of apoptosis by immunohistochemistry to active caspase-3, active caspase-7, or cleaved parp in monolayer cells and spheroid and subcutaneous xenografts of human carcinoma. *Journal of Histochemistry & Cytochemistry*, 57(4):289–300, 2009.

N. Brijwani, M. Jain, M. Dhandapani, F. Zahed, P. Mukhopadhyay, M. Biswas, D. Khatri, V. D. Radhakrishna, B. Majumder, P. Radhakrishnan, et al. Rationally co-targeting divergent pathways in kras wild-type colorectal cancers by canscript technology reveals tumor dependence on notch and erbb2. *Scientific reports*, 7(1):1–12, 2017.

P.-L. Chen, W. Roh, A. Reuben, Z. A. Cooper, C. N. Spencer, P. A. Prieto, J. P. Miller, R. L. Bassett, V. Gopalakrishnan, K. Wani, et al. Analysis of immune signatures in longitudinal tumor samples yields insight into biomarkers of response and mechanisms of resistance to immune checkpoint blockade. *Cancer discovery*, 6(8):827–837, 2016.

S. Diehl and M. Rincón. The two faces of il-6 on th1/th2 differentiation. *Molecular immunology*, 39(9): 531–536, 2002.

M. A. Fishman and A. S. Perelson. Th1/th2 cross regulation. *Journal of theoretical biology*, 170(1): 25–56, 1994.

M. A. Fishman and A. S. Perelson. Th1/th2 differentiation and cross-regulation. *Bulletin of mathematical biology*, 61(3):403–436, 1999.

G. J. Freeman, E. J. Wherry, R. Ahmed, and A. H. Sharpe. Reinvigorating exhausted hiv-specific t cells via pd-1–pd-1 ligand blockade. *Journal of Experimental Medicine*, 203(10):2223–2227, 2006.

X. Lai and A. Friedman. Combination therapy of cancer with cancer vaccine and immune checkpoint inhibitors: A mathematical model. *PLoS One*, 12(5):e0178479, 2017.

W. Lasek, R. Zagożdżon, and M. Jakobisiak. Interleukin 12: still a promising candidate for tumor immunotherapy? *Cancer Immunology, Immunotherapy*, 63(5):419–435, 2014.

S. E. Macatonia, N. A. Hosken, M. Litton, P. Vieira, C.-S. Hsieh, J. A. Culpepper, M. Wysocka, G. Trinchieri, K. M. Murphy, and A. O'Garra. Dendritic cells produce il-12 and direct the development of th1 cells from naive cd4+ t cells. *The Journal of Immunology*, 154(10):5071–5079, 1995.

B. Majumder, U. Baraneedharan, S. Thiyagarajan, P. Radhakrishnan, H. Narasimhan, M. Dhandapani, N. Brijwani, D. D. Pinto, A. Prasath, B. U. Shanthappa, et al. Predicting clinical response to anticancer drugs using an ex vivo platform that captures tumour heterogeneity. *Nature communications*, 6(1): 1–14, 2015.

M. D. McKay, R. J. Beckman, and W. J. Conover. Comparison of three methods for selecting values of input variables in the analysis of output from a computer code. *Technometrics*, 21(2):239–245, 1979.

B. F. Morel, J. Kalagnanam, and P. A. Morel. Mathematical modeling of th1-th2 dynamics. In *Theoretical and experimental insights into immunology*, pages 171–190. Springer, 1992.

T. Okazaki and T. Honjo. The pd-1–pd-l pathway in immunological tolerance. *Trends in immunology*, 27 (4):195–201, 2006.

N. Riaz, J. J. Havel, V. Makarov, A. Desrichard, W. J. Urba, J. S. Sims, F. S. Hodi, S. Martín-Algarra, R. Mandal, W. H. Sharfman, et al. Tumor and microenvironment evolution during immunotherapy with nivolumab. *Cell*, 171(4):934–949, 2017.

J. P. Ridge, F. Di Rosa, and P. Matzinger. A conditioned dendritic cell can be a temporal bridge between a cd4+ t-helper and a t-killer cell. *Nature*, 393(6684):474, 1998.

M. Rincón, J. Anguita, T. Nakamura, E. Fikrig, and R. A. Flavell. Interleukin (il)-6 directs the differentiation of il-4–producing cd4+ t cells. *Journal of Experimental Medicine*, 185(3):461–470, 1997.

S. Romagnani. The th1/th2 paradigm. *Immunology today*, 18(6):263–266, 1997.

S. Sakaguchi. Regulatory t cells: key controllers of immunologic self-tolerance. *Cell*, 101(5):455–458, 2000.

M. Sznol and L. Chen. Antagonist antibodies to pd-1 and b7-h1 (pd-l1) in the treatment of advanced human cancerresponse. *Clinical Cancer Research*, 19(19):5542–5542, 2013.

J. A. Trapani and M. J. Smyth. Functional significance of the perforin/granzyme cell death pathway. *Nature Reviews Immunology*, 2(10):735, 2002.

V. Vaira, G. Fedele, S. Pyne, E. Fasoli, G. Zadra, D. Bailey, E. Snyder, A. Faversani, G. Coggi, R. Flavin, et al. Preclinical model of organotypic culture for pharmacodynamic profiling of human tumors. *Proceedings of the National Academy of Sciences*, 107(18):8352–8356, 2010.

A. Yates, C. Bergmann, J. L. Van Hemmen, J. Stark, and R. Callard. Cytokine-modulated regulation of helper t cell populations. *Journal of theoretical biology*, 206(4):539–560, 2000.
